# Visualizing the human olfactory projection and ancillary structures in a 3D reconstruction

**DOI:** 10.1038/s42003-024-07017-4

**Published:** 2024-11-08

**Authors:** Victoria F. Low, Chinchien Lin, Shan Su, Mahyar Osanlouy, Mona Khan, Soroush Safaei, Gonzalo Maso Talou, Maurice A. Curtis, Peter Mombaerts

**Affiliations:** 1 Department of Anatomy and Medical Imaging, The University of Auckland, Auckland, New Zealand; 2 Auckland Bioengineering Institute, The University of Auckland, Auckland, New Zealand; 3 Max Planck Research Unit for Neurogenetics, Frankfurt, Germany

**Keywords:** Olfactory bulb, Olfactory bulb

## Abstract

Visualizing in 3D the histological microanatomy of the human olfactory projection from the olfactory mucosa in the nasal cavity to the olfactory bulbs in the cranial cavity necessitates a workflow for handling a great many sections. Here, we assembled a 3D reconstruction of a 7.45 cm^3^ *en-bloc* specimen extracted from an embalmed human cadaver. A series of 10 µm coronal sections was stained with quadruple fluorescence histology and scanned in four channels. A trained anatomist manually segmented six structures of interest in a subset of the sections to generate the ground truth. Six convolutional neural networks were then trained for automatic segmentation of these structures in 1234 sections. A high-performance computing solution was engineered to register the sections based on the fluorescence signal and segmented structures. The resulting 3D visualization offers several novel didactic opportunities of interactive exploration and virtual manipulation. By extrapolating manual counts of OSNs in a subset of sections to the calculated volume of the envelope of the entire olfactory epithelium, we computed a total of ~2.7 million OSNs in the specimen. Such empirically derived information helps assess the extent to which the organizational principles of the human olfactory projection may differ from those in mice.

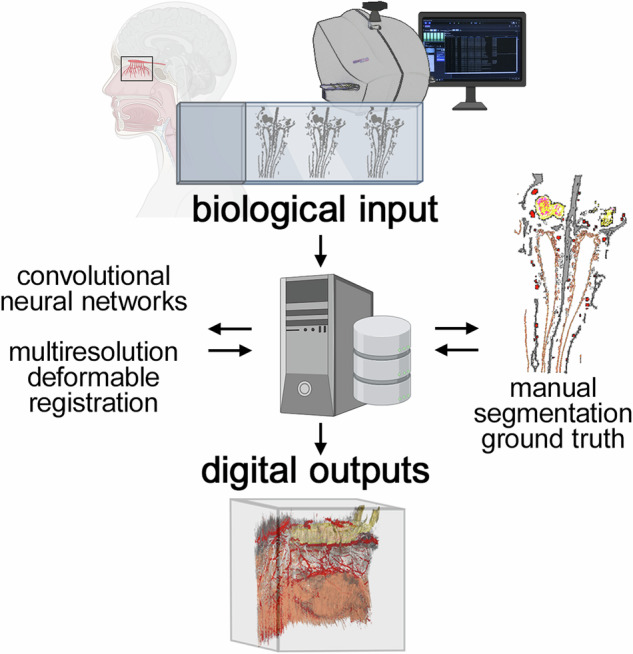

## Abstract (web summary)

The microanatomy of the axonal projections of olfactory sensory neurons from the olfactory epithelium to the olfactory bulb in a human enbloc specimen is visualized in a 3D reconstruction assembled by deep learning and high performance computing.

## Introduction

The human sense of smell has recently become the subject of intense popular and scientific interest due to the frequent association of olfactory dysfunction with SARS-CoV-2 infection^1^, often persisting long after COVID-19 symptoms^2^. Olfactory dysfunction presents in almost all patients with Parkinson’s disease^3,4^ and Alzheimer’s disease^5–7^ several years prior to the onset of motor and cognitive impairments, together with pathological changes in the olfactory bulb^8–10^. The sense of smell can be seen as serving as a sensitive sentinel for these two common neurodegenerative diseases. Olfactory dysfunction is also correlated with mortality^11^ and heart disease^12^. But, complicating matters, the human sense of smell declines naturally with age^13^. Current concepts about the human sense of smell are largely derived from studies on laboratory mice and rats. Paling in comparison with the blooming literature on genetically engineered mice in recent decades is the snail’s pace of the primary research literature on the histological microanatomy of the human olfactory projection. It remains unclear to which extent the organizational principles of this microanatomy in humans resemble those in mice.

**Fig. 1:**
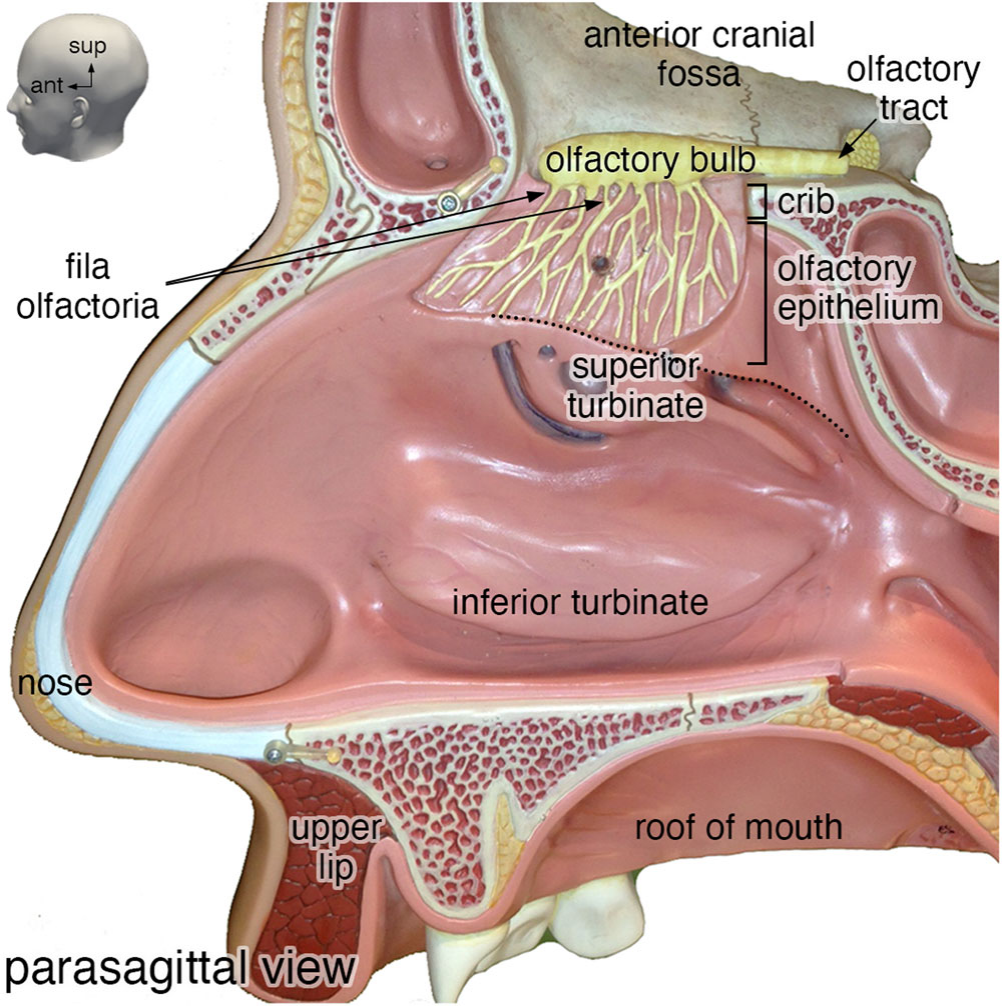
Photograph of an anatomical teaching model of a human head. Parasagittal view of the lateral wall of the right nasal cavity from a SOMSO® anatomical teaching model showing the position of the olfactory bulb on the skull base and the projection of fila olfactoria through the cribriform plate (crib). ant, anterior; sup, superior.

The olfactory projection consists of olfactory sensory neurons (OSNs) projecting their axons from the olfactory mucosa in the nasal cavity to the olfactory bulbs in the cranial cavity (Fig. 1)^14–16^. The olfactory mucosa comprises the olfactory epithelium and the underlying lamina propria, separated by a basal lamina^17–20^. Its surface area is estimated grossly at ~500 mm^2^ in humans^21^, amounting to a few percent of the surface area of the nasal mucosa^22^, the majority of which is respiratory mucosa. A mature OSN extends a single dendrite apically to the lumen of the nasal cavity. On the dendritic cilia, odorant receptors^23^ interact with odorous molecules from the inhaled air. A mature OSN projects a single unmyelinated axon from the olfactory epithelium through the basal lamina into the lamina propria where it fasciculates with other OSN axons into olfactory axon fascicles of progressively larger diameter^24^. These fascicles traverse the cribriform plate^25^ ipsilaterally as macroscopically visible fila olfactoria^26,27^, which together make up the olfactory nerve, the first cranial nerve^28,29^. The fila olfactoria innervate the glomerular array in the ipsilateral olfactory bulb. Within glomeruli, OSN axons terminate and synapse with the dendrites of the second-order neurons in the olfactory pathway. Immunohistochemical staining patterns of the human olfactory epithelium are similar to those in mice and rats^30^. Immunohistochemical studies of the human olfactory bulb have revealed basic characteristics similar to mice and rats^31–34^ and the human interneuron populations are also in general agreement with those in mice and rats^35^. But these similarities may be superficial, possibly even deceptive.

Striking differences raise questions about extrapolating current concepts about olfaction from mice to humans. For instance, a mouse glomerulus receives axonal input from OSNs that express the same odorant receptor gene^36–38^ and are located in one of several zones of the olfactory epithelium^39^. A mature mouse OSN expresses a single odorant receptor gene^40,41^. With ~3600 glomeruli in a mouse olfactory bulb^42^ and 1141 intact odorant receptor genes in the mouse genome^43^, OSNs expressing a given odorant receptor gene project their axons on average to ~3 glomeruli per olfactory bulb. However, the neat arrangement of the glomerular array seen in mice and rats is missing in humans. Glomeruli in the human olfactory bulb exhibit a broad range of shapes and sizes, precluding reliable counts of glomeruli. One study came up with 2975–9325 glomeruli in a human olfactory bulb^33^. With 389 intact odorant receptor genes in the human genome^43^, OSNs expressing a given odorant receptor gene would thus project on average to ~8–24 glomeruli, if the organizational principles are the same as in mice.

The human olfactory projection can be visualized with a range of imaging techniques at various levels of resolution such as magnetic resonance imaging, computed tomography, and micro-computed tomography, but these techniques do not have microscopic resolution and do not afford cell type-specific labeling with antibodies or other markers. Much of the body of knowledge about the histological microanatomy of the human olfactory projection comes from relatively small biopsies procured from living subjects or from specimens harvested at autopsy. In the literature, typically images are shown of a few fields of view from a few stained sections photographed under a microscope. The magnitude of the sampling error in these approaches is not known. With the advent of high-resolution fluorescence whole-slide scanning in multiple channels, histological datasets can now be processed by deep-learning techniques to automatically obtain a delineation or annotation (“segmentation”) of structures of interest in a great many sections. Convolutional neural networks (CNNs), in particular U-Net^44^, are considered the standard to perform deep-learning based supervised automatic segmentation: a CNN is trained to segment a structure of interest based on a finite number of segmentations performed manually by an expert human observer generating the “ground truth”.

Here, we combined quadruple fluorescence histological staining with deep learning (for segmentation) and high-performance computing (for registration) to assemble a 3D reconstruction of a specimen extracted from an embalmed human cadaver. Following manual segmentation by an expert, automatic segmentation was performed by CNNs. The computationally-expensive registration of the images was performed by a high-performance computing cluster.

## Results

### Screening of *en-bloc* specimens extracted from embalmed human cadavers

**Fig. 2:**
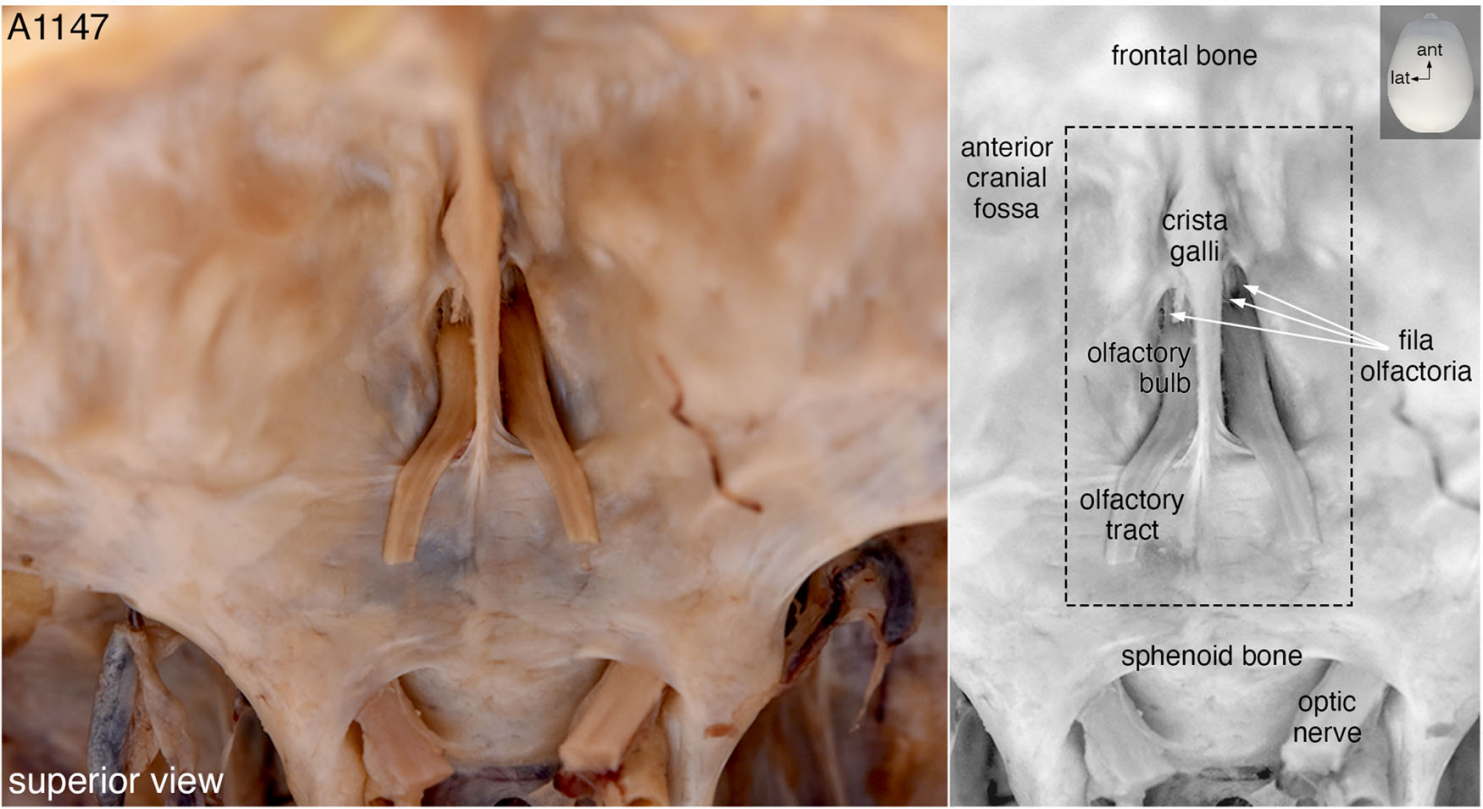
The skull base of case A1147. Superior view of the anterior cranial fossa showing the olfactory bulbs attached to the cribriform plate and the parts of the olfactory tracts that remained after the bulk of the brain had been removed. In a gray-scale duplicate image of the color photo, three fila olfactoria are indicated with white arrows and the black dashed box approximates where the incisions were made to extract the quadrangular *en-bloc* specimen. The left side of the images corresponds to the left side of the head. ant, anterior; lat, lateral.

We extracted an *en-bloc* specimen containing the olfactory projection and ancillary structures of bone and vasculature from 6 human cadavers that were each flushed with 5 liters of saline solution and then embalmed with 20 liters of Dodge Anatomical Arterial Michigan Mixture. This embalming fluid is commonly used for cadavers in medical teaching. The extraction entailed making incisions in the anterior cranial fossa in the form of a quadrangle and extends inferiorly to encompass the superior turbinates (Fig. 2), similar to techniques reported by others^18,45–48^. These specimens consist of a complex 3D configuration of soft and hard tissues, including a part of the anterior skull base and a portion of the nasal cavity, filled with air. After EDTA-mediated decalcification and embedding in paraffin, the six specimens were each cut into a series of 10 µm coronal sections, yielding a grand total of 11,805 sections. Tissue quality was assessed on every 150th section by chromogenic staining and imaging under a brightfield microscope (Supplementary Fig. 1). The specimen of case A1147, which had been cut into 1923 sections, displayed the best structural preservation and immunoreactivity. Anticipating a labor-intensive and computationally-expensive workflow, we judged A1147 among the six specimens to be of sufficient quality for assembling a 3D reconstruction. This specimen, henceforth referred to as “the specimen”, measured ~1.92 cm in the anterior-posterior dimension, ~2.27 cm superior-inferior, and ~1.71 cm left-right, corresponding to a volume of ~7.45 cm^3^.

### Quadruple fluorescence histological staining and scanning

After tissue quality assessment and further validation, 1851 sections mounted on 617 slides remained of the specimen. Next, these sections were subjected to quadruple fluorescence histological staining for Hoechst 33342 (blue), *Ulex europaeus* agglutinin-1 (UEA1) (green), olfactory marker protein (OMP) (red), and vesicular glutamate transporter-2 (VGLUT2) (far-red).

Hoechst 33342, henceforth referred to as “Hoechst”, is a fluorescent dye that stains DNA and thereby identifies the nuclei of all cells. UEA1 is a plant lectin that binds to many glycoproteins and glycolipids containing α-linked fucose residues and has been widely used as a broad marker in histological studies. UEA1 stains mature and immature OSNs in rat^49^ and human^50^ as well as basal cells in the olfactory and respiratory epithelium. Biotinylated UEA1 was detected with fluorescently labeled streptavidin. Immunochemistry visualized OMP, which has traditionally been used as a marker of the olfactory projection^51^. This cytoplasmic protein is highly expressed in maturing human OSNs in all their subcellular compartments, from the dendrites all the way to the axon terminals within the glomeruli of the olfactory bulb^31,32,52^. Lastly, immunochemistry visualized VGLUT2, a marker of OSN axon terminals within the glomeruli^33,34^. The rationale of our staining strategy was to inform the CNNs with a palette of fluorescent signals with various levels of specificity, from generic (Hoechst) over broad (UEA1) to highly specific (OMP and VGLUT2).

**Fig. 3:**
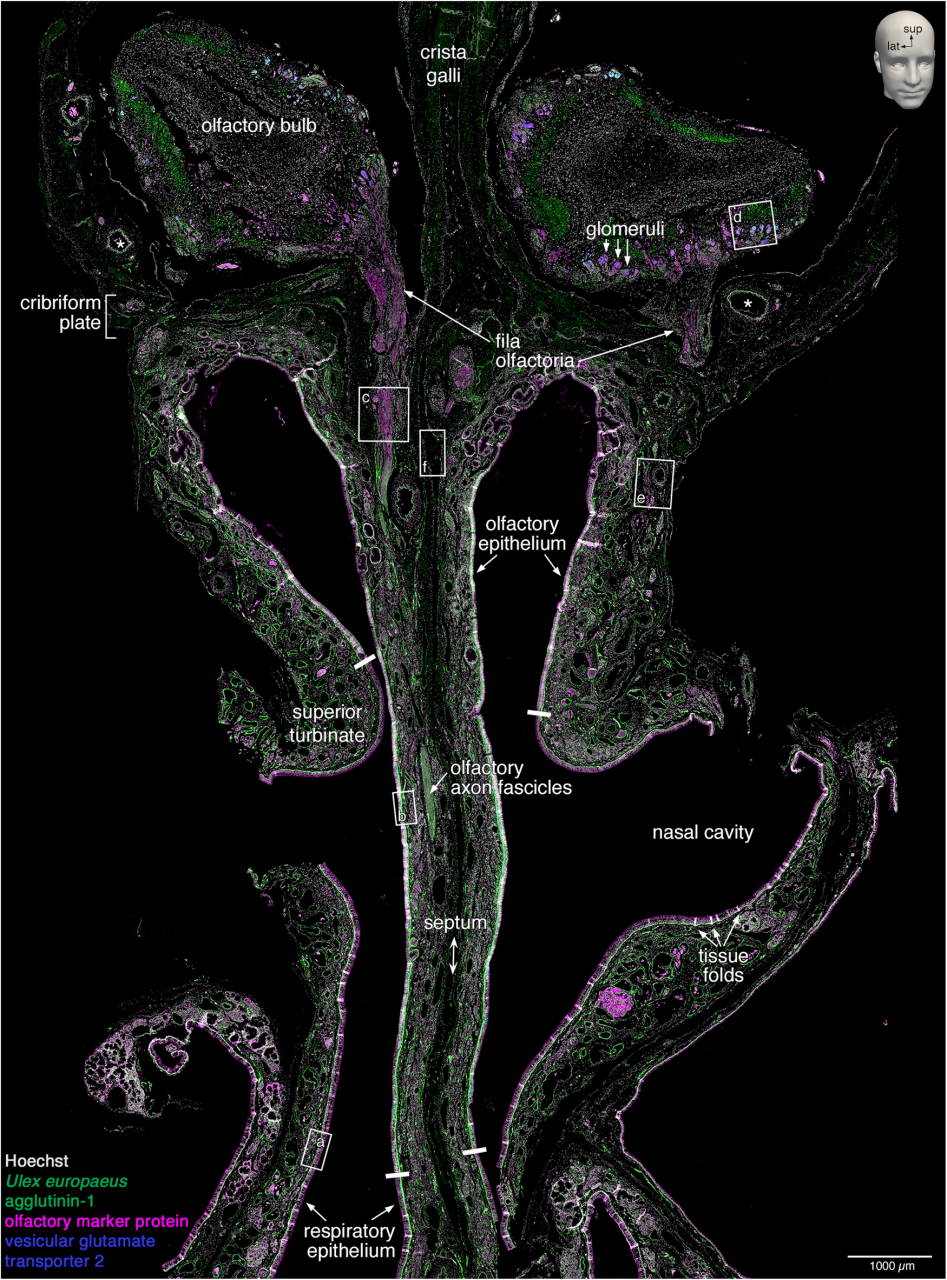
Scanned image of a quadruple-stained coronal section. A representative 10 µm coronal section (#1057) stained fluorescently for Hoechst (gray), *Ulex europaeus* agglutinin-1 (green), olfactory marker protein (magenta), and vesicular glutamate transporter 2 (blue). The image was taken on a PANNORAMIC MIDI II fluorescence digital slide scanner as an extended-focus three-level z-stack. The dimensions of the image are ~1.0 cm wide by ~1.5 cm long. The perpendicular plate of the ethmoid bone (septum) divides the left and right nasal cavities, and the upper part of the perpendicular plate (crista galli) separates the two olfactory bulbs. The nasal cavity is lined by nasal epithelium, the majority of which is respiratory epithelium. The dorsal-superior aspect of the nasal cavity harbors olfactory epithelium, as defined by the presence of OSNs. On the lateral aspect, the olfactory epithelium covers the superior turbinate, and on the septal aspect, it extends below the position equivalent to the curvature of the superior turbinate. Four thick white bars indicate the inferior boundaries of the olfactory epithelium on the superior turbinates and the septum. Within the lamina propria, axons of OSNs group into olfactory axon fascicles of progressively larger diameter that form macroscopically visible fila olfactoria, which traverse ipsilaterally the cribriform plate toward the ipsilateral olfactory bulb. The lamina propria also contains a dense network of arterial vasculature (two asterisks indicate accessory olfactory arteries). Rectangles (a–f) are displayed at higher magnification in Fig. 4a–f; the position of the letter informs the orientation of the high-magnification view. Small tissue folds in the nasal mucosa, mostly in the epithelium, appear as bright white stripe-like patches. The left side of the image corresponds to the right side of the head. lat, lateral; sup, superior.

Slides were scanned in four channels using a PANNORAMIC MIDI II fluorescence digital slide scanner to obtain a three-level z-stack, which was consolidated into an extended focus. The 1851 sections of the specimen took ~2000 h of scanning time and 1234 sections (66.67%) were of suitable quality to be incorporated into the 3D reconstruction. Henceforth, these 1234 sections are referred to as the “complete dataset”. Supplementary Fig. 2 shows the location of the sections that were not available or excluded, and sections that were excluded based on a given criterion. Figure 3 shows a scanned image of a representative section, with annotations of the relevant microanatomical structures.

### Histology of the human olfactory projection and ancillary structures

The file size of the complete dataset (~2.9 TB) required us to make a selection among the many structures that could in principle be segmented based on the information from the quadruple fluorescence staining. We were interested in six structures, which are henceforth called “structures of interest”: nasal epithelium, OSNs, glomeruli, olfactory bulbs, vasculature (arteries), and bone. The nasal epithelium consists of the most part of respiratory and olfactory epithelium and lines the air-filled lumen of the nasal cavity. The olfactory bulbs can be outlined readily by their anatomical shape and Hoechst staining.

**Fig. 4:**
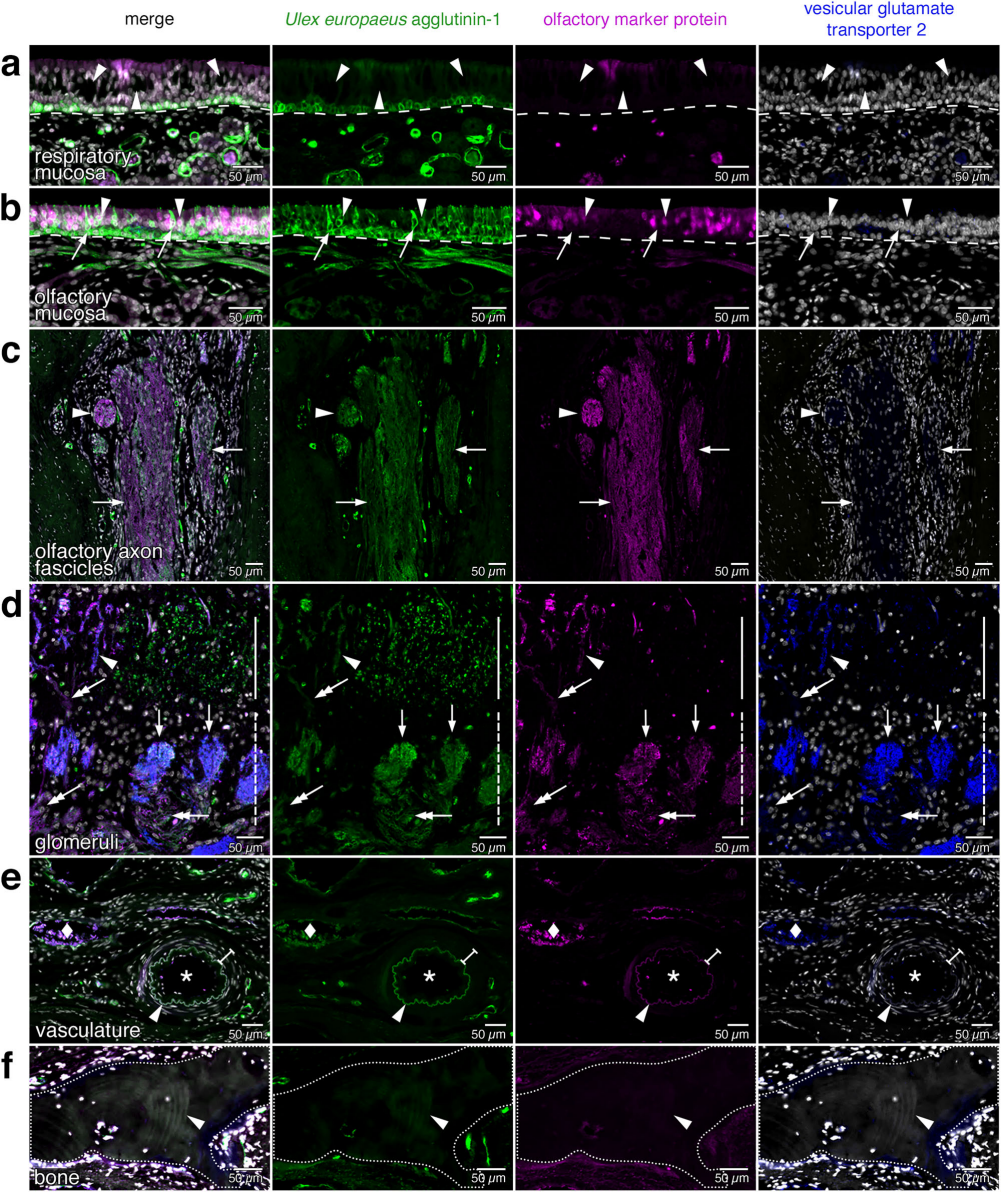
Histology of the olfactory projection and ancillary structures. High-magnification views of the rectangles (**a**–**f**) demarcated in Fig. 3. **a** The respiratory mucosa consists of respiratory epithelium and lamina propria separated by a basal lamina (dashed line). Dark spaces reflect goblet cells (arrowheads). The faint signal for OMP near the middle of the image is an artifact produced by a tissue fold. **b** The olfactory mucosa consists of olfactory epithelium and lamina propria separated by a basal lamina (dashed line). The olfactory epithelium contains immature OSNs (arrows) and mature, OMP-immunoreactive OSNs (arrowheads). **c** Olfactory axon fascicles, formed by OSN axons within the lamina propria, appear as longitudinal or oblique sectional views (arrows) or as a cross-sectional view (arrowhead). **d** Glomeruli (arrows) are innervated by axons of OSNs (double arrows). Most glomeruli reside within the glomerular layer (dashed vertical line). Some invasive glomeruli with an elongated appearance (arrowhead) reside deeper, within the external plexiform layer (solid vertical line). **e** Arterial vasculature is identified by an internal elastic lamina (arrowhead) and a surrounding layer of elongated nuclei of smooth muscle cells (I-beam line) that are parallel to the internal elastic lamina and surround a luminal space (asterisk). The diamond indicates a vein. **f** Bone (dotted line) is acellular, has a laminar appearance (arrowhead), and is not fluorescently stained.

Figure 4 shows high-magnification views of the rectangles demarcated in Fig. 3. The respiratory epithelium lacks OSNs and makes up the respiratory mucosa together with the underlying lamina propria (Fig. 4a). The olfactory epithelium and the underlying lamina propria constitute the olfactory mucosa, and OSNs can be identified by combining OMP immunoreactivity and UEA1 staining (Fig. 4b). Axons of OSNs fasciculate into olfactory axon fascicles within the lamina propria (Fig. 4c). Glomeruli are irregularly shaped structures mostly located near the surface of the olfactory bulb, and OSN axon terminals within the glomeruli can be identified with VGLUT2 immunoreactivity (Fig. 4d). Vasculature (arteries) can be identified by UEA1 staining of the internal elastic lamina and Hoechst staining of the elongated nuclei of smooth muscle cells surrounding a luminal space (Fig. 4e). There is no vasculature within the olfactory epithelium. Bone has a laminar appearance and is devoid of nuclei (Fig. 4f).

### Manual segmentation generates the ground truth

The next phase entailed the manual segmentation of the six structures of interest by a trained anatomist, relying on shape and context of location, and aided by the palette of fluorescence signals. Manual segmentation was carried out in 26–57 sections and took ~1300 h. Simple and high-contrast structures of interest such as vasculature took ~2 h of manual segmentation per section, while complex structures of interest that spanned a large area such as OSNs took ~12–16 h per section. Manual segmentation of the six structures of interest generated an expert reference, called the ground truth.

The nasal epithelium is a pseudostratified columnar epithelium and was segmented based on the dense organized arrangement of nuclei of ciliated respiratory cells, goblet cells, OSNs, sustentacular cells, and basal cells, as well as the basal lamina, which separates the epithelium from the lamina propria. Mature and immature OSNs were segmented together by combining information from the OMP immunoreactivity and UEA1 staining. When OSNs were sparse, they could be segmented at single-cell resolution, but when they were densely packed, they were segmented as groups. Therefore, as a whole, the OSN segmentation does not have single-OSN resolution. Of note, the OSN segmentation encompasses OSNs across their entire length of several cm: from the dendrites and cell bodies within the nasal epithelium, along the axonal trajectory within the lamina propria, through the cribriform plate, all the way to the axon terminals within glomeruli. The intersection of the nasal epithelium segmentation and the OSN segmentation defines the olfactory epithelium, which was however not segmented as such. Human glomeruli cannot be identified reliably as discrete structures and there are numerous atypical glomerular-like structures^34^. Therefore, as a whole, the glomerulus segmentation does not have single-glomerulus resolution, precluding attempts to directly count the total number of glomeruli. The glomerulus segmentation ought to be seen as the segmentation of the glomerular component of the olfactory bulb.

### Training convolutional neural networks for automatic segmentation

After obtaining the ground truth, six U-Net CNNs^44^ were trained for automatic segmentation of the six corresponding structures of interest. A graphics processing unit (GPU) cluster with 10 NVIDIA Tesla V100 32 GB was used to train one CNN per GPU. During the training process, a CNN receives as input the four channels of a subset of sections together with the paired ground truth from the training dataset. The CNN learns to automatically generate the output segmentation by combining the inputs, mainly by convolution operations at different resolutions. Regions without fluorescence signal help delineate the background pixels and thereby define the boundaries of the foreground pixels, for instance, for nasal epithelium (lining the empty space of the lumen of the nasal cavity) and for vasculature (surrounding empty space, as most of the blood was removed by flushing and embalming the cadaver). Hoechst and UEA1 are markers with a generic and broad staining pattern respectively, and the OMP and VGLUT2 immunoreactive signals mark a specific cell type and subcellular compartment respectively.

Initially, six consecutive sections from four regions (a total of 24 sections) evenly spaced throughout the specimen were manually segmented for all six structures of interest (Supplementary Fig. 2). Five sections with the paired ground truth from each of these four regions were used for training (the “training dataset”, a total of 20 sections) and one section for validation (the “validation dataset”, a total of 4 sections). To monitor the performance of the CNNs during training, the Dice similarity coefficient (DSC) and binary cross-entropy were calculated on the validation dataset. The DSC, an intuitive measure of agreement between segmentations, is the ratio of the intersection between the CNN segmentation and the ground truth over their union, i.e., the degree of overlap, and ranges from 0 to 1 (the higher the value, the better, with 1 representing perfect agreement). The binary cross-entropy is a metric from information theory and is inversely related to the agreement between the CNN segmentation and the ground truth (the lower the value, the better, with 0 representing perfect agreement). A bootstrap approach was employed to iteratively improve the DSC and binary cross-entropy of the CNNs. We judged the CNN segmentations on unseen sections (i.e., sections that were not part of the training dataset and of the validation dataset) by overlaying them with the corresponding sections and visually checking the accuracy of the structures segmented. We manually corrected the sections with the lower-quality CNN segmentations and added them to the training dataset, which was then used to further train the CNN. This process was repeated until a DSC greater than 0.85 was achieved or there were diminishing returns.

**Fig. 5:**
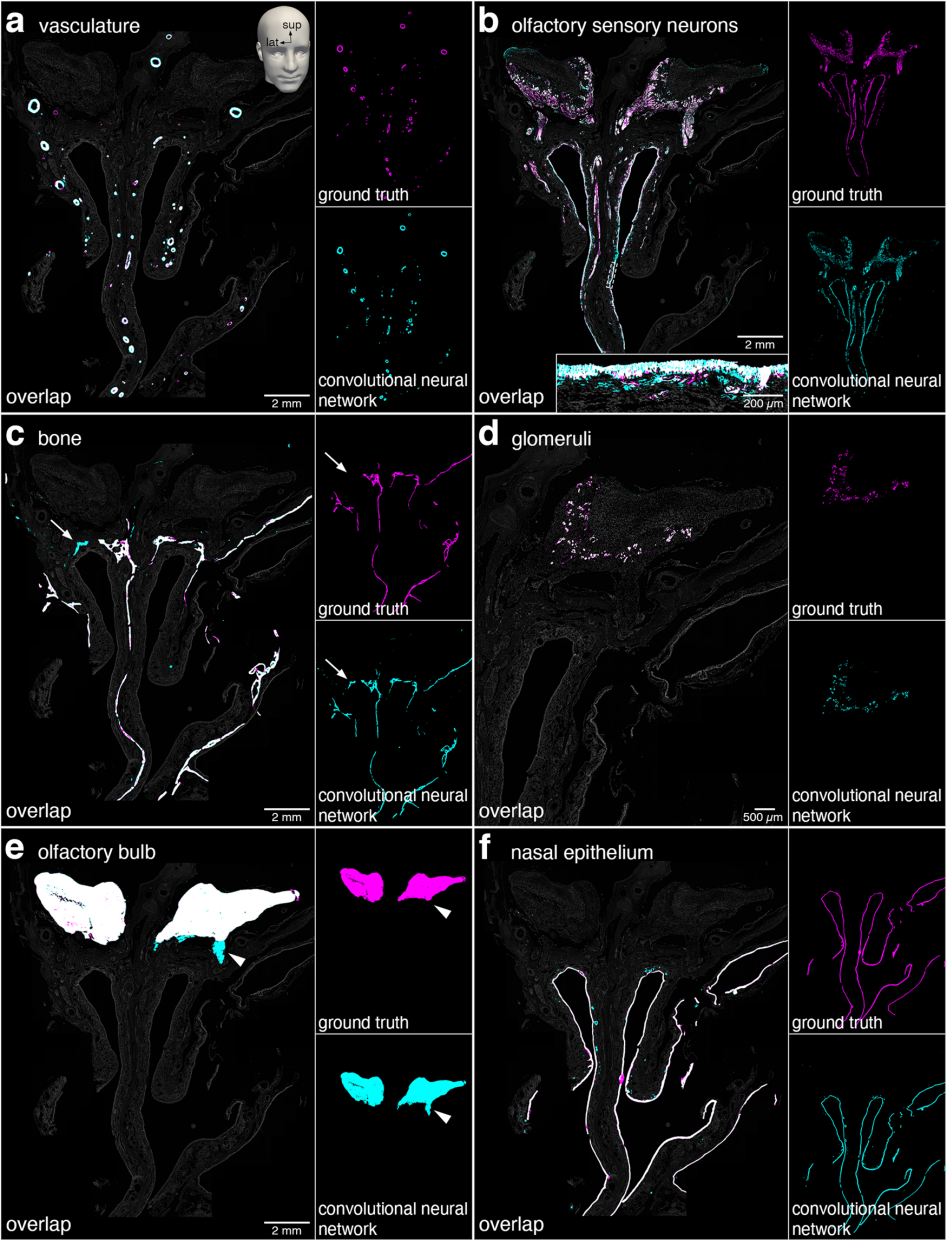
Comparison of the ground truth and convolutional neuronal network segmentations. The overlap (white) represents the agreement between the ground truth segmentation (magenta) and the CNN segmentation (cyan) in testing section #1401. The segmentations are overlayed on the corresponding tissue (gray). Cyan represents an overprediction (false-positive) and magenta an underprediction (false-negative) by the CNN segmentation. The six structures of interest are ordered in the decreasing number of sections used to train and validate the CNN. **a** Vasculature segmentation (DSC = 0.808, binary cross-entropy = 0.0148). **b** OSN segmentation (DSC = 0.760, binary cross-entropy = 0.0173). The inset shows a higher magnification of the overlap at the level of the olfactory epithelium: finer details, including OSN dendrites and axons, are not detected properly, resulting in an overprediction in the CNN segmentation (more cyan) in comparison to the ground truth (white and magenta). **c** Bone segmentation (DSC = 0.885, binary cross-entropy = 0.0121). An area of bone (white arrow) was not segmented in the ground truth due to human error but picked up by the CNN. **d** Glomerulus segmentation (DSC = 0.779, binary cross-entropy = 0.0017). A part of the left olfactory bulb is shown. **e** Olfactory bulb segmentation (DSC = 0.967, binary cross-entropy = 0.0044). An area of the olfactory tract was detected by the CNN (white arrowhead). **f** Nasal epithelium segmentation (DSC = 0.927, binary cross-entropy = 0.0053).

Different numbers of sections were used for training and validation of the six CNNs for the six structures of interest (Supplementary Fig. 3). The CNN for vasculature required the greatest number of sections for training (45) and validation (10) (Fig. 5a). The CNN for OSNs was trained with 38 and validated against 6 sections (Fig. 5b). Dendrites and axons of OSNs were not resolved to the same high resolution as in the ground truth particularly when many OSNs were packed together, resulting in systematic overprediction of the CNN segmentations for OSNs (inset of Fig. 5b, Supplementary Fig. 4). The CNN for bone did not perform well initially—bone was missing and non-specific structures were included—but did perform satisfactorily after further training (Fig. 5c). The CNN for glomeruli performed well with a training dataset of 25 sections and a validation dataset of 5 sections (Fig. 5d). Lastly, the CNNs for the olfactory bulb (Fig. 5e) and the nasal epithelium (Fig. 5f) performed well with the initial training dataset of 20 sections and the validation dataset of 4 sections.

After the CNNs had been trained, the same 10 NVIDIA Tesla V100 32 GB configuration performed the automatic segmentations of the six structures of interest for each of the 1234 sections of the complete dataset.

After the complete dataset was segmented, a “sanity check” was carried out by a trained anatomist assessing whether artifacts or aberrant anatomical structures had been generated. Finally, the CNNs were tested on two additional manually segmented sections (#850 and #1401), the “testing dataset” (Supplementary Fig. 2). These two sections were unseen by the CNNs during training and validation; were randomly selected among sections that contained all six structures of interest; and had not been used previously for any other purpose. The CNN segmentations of these two sections were unknown to the trained anatomist who had generated the ground truth segmentations. The resulting DSC and binary cross-entropy values suggest that the CNNs were not overfitted to the training and validation datasets (Supplementary Fig. 3).

### Image registration and visualization in 3D

The alignment of the images, called the “registration”, was the most computationally-expensive phase and required a memory allocation in the order of hundreds of GB. To this end, we made use of a high-performance computing cluster at the New Zealand eScience Infrastructure. A total of 1082 central processing unit (CPU) hours from a cluster with 96 Intel Xeon Gold 6136 @ 3.00 GHz (Skylake) was used to perform a multiresolution, deformable registration of the 1234 sections, aligning the six structures of interest using the four fluorescence channels.

The registration operations were performed by using the C++ SimpleElastix library^53^ and involved two sequential steps: an intra-block registration, during which 247 blocks of five contiguous sections were registered independently, followed by an inter-block registration, during which the interfaces between two consecutive blocks of five sections were registered in finer detail to yield a smooth alignment. This strategy has two advantages over a direct registration of the complete dataset, which would entail performing a registration of each section with the previous section. First, the intra-block registration can be executed in parallel for all blocks, reducing the total time elapsed. Second, the inter-block registration copes adequately with the artifact of the so-called banana effect^54^ by performing an interpolation of the registration between comparable sections of adjacent blocks, thereby smoothening the registration, with less distorted transition.

Finally, we displayed the 1234 registered sections as a 3D reconstruction by converting the files into a format that can be visualized in 3D with ParaView^55,56^ (.nifti) and Neuroglancer^57^ (neuroglancer precomputed format). The Neuroglancer files were downsampled 16-fold to allow for fluent visualization in a web browser.

**Fig. 6:**
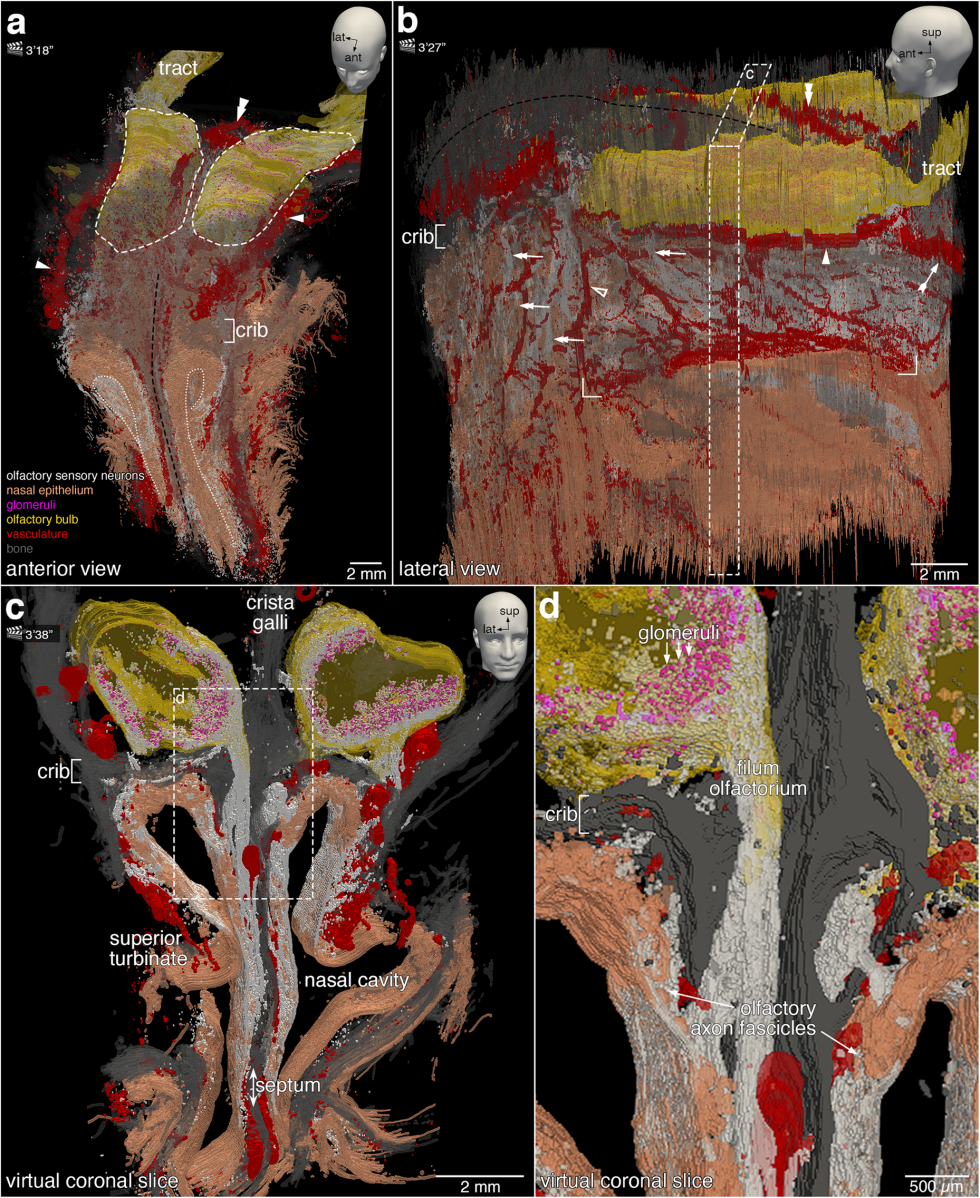
Still views of the 3D reconstruction. The head models with positional indicators of anterior (ant), lateral (lat), and superior (sup) in the top right corners indicate the angle of the still views on the 3D construction. The corresponding time points in Supplementary Movie 1 are indicated next to the clapperboards, as minutes and seconds. Images were taken in ParaView. **a** Anterior-superior still view showing OSNs (white) and the nasal epithelium (orange). At the anterior face of the specimen, the nasal epithelium was physically cut. The apical side of the nasal epithelium is contoured by a white dotted line. The olfactory projection is supported by the ethmoid bone (gray), which includes the cribriform plate (crib) and the nasal septum (black-dashed line), and by a dense arterial vasculature (red). The proper olfactory artery (double arrowhead) and the accessory olfactory arteries (arrowheads) are indicated. The olfactory bulbs (yellow, with the contours indicated by a white dashed line) are partially obfuscated by bone. The olfactory tract (tract) extends posteriorly from the olfactory bulb. **b** Lateral still view revealing the arterial vasculature (red): the proper olfactory artery (double arrowhead), accessory olfactory artery (arrowhead), anterior ethmoidal artery (hollow arrowhead), posterior ethmoidal artery (feathered arrow), and the branches of the sphenopalatine artery (rectangular brackets). A network of smaller branches of the anterior and posterior ethmoidal arteries are observed along with the olfactory axon fascicles (double arrows). The crista galli (black dashed line) separates the olfactory bulbs. The area demarcated with a white dashed line and annotated with the letter c is the virtual coronal slice shown in (**c**). **c** The ~1 mm-thick virtual coronal slice viewed in a coronal plane reveals the microanatomical relationships between the nasal cavity and the olfactory bulbs. OSNs reside in the superior aspect of the nasal epithelium. **d** A high-magnification view of the dashed rectangle demarcated in (**c**). Axons of OSNs form within the lamina propria olfactory axon fascicles of progressively larger diameter. The fila olfactoria traverse ipsilaterally the cribriform plate (crib) toward the ipsilateral olfactory bulb. OSN axons terminate within glomeruli (magenta).

**Fig. 7:**
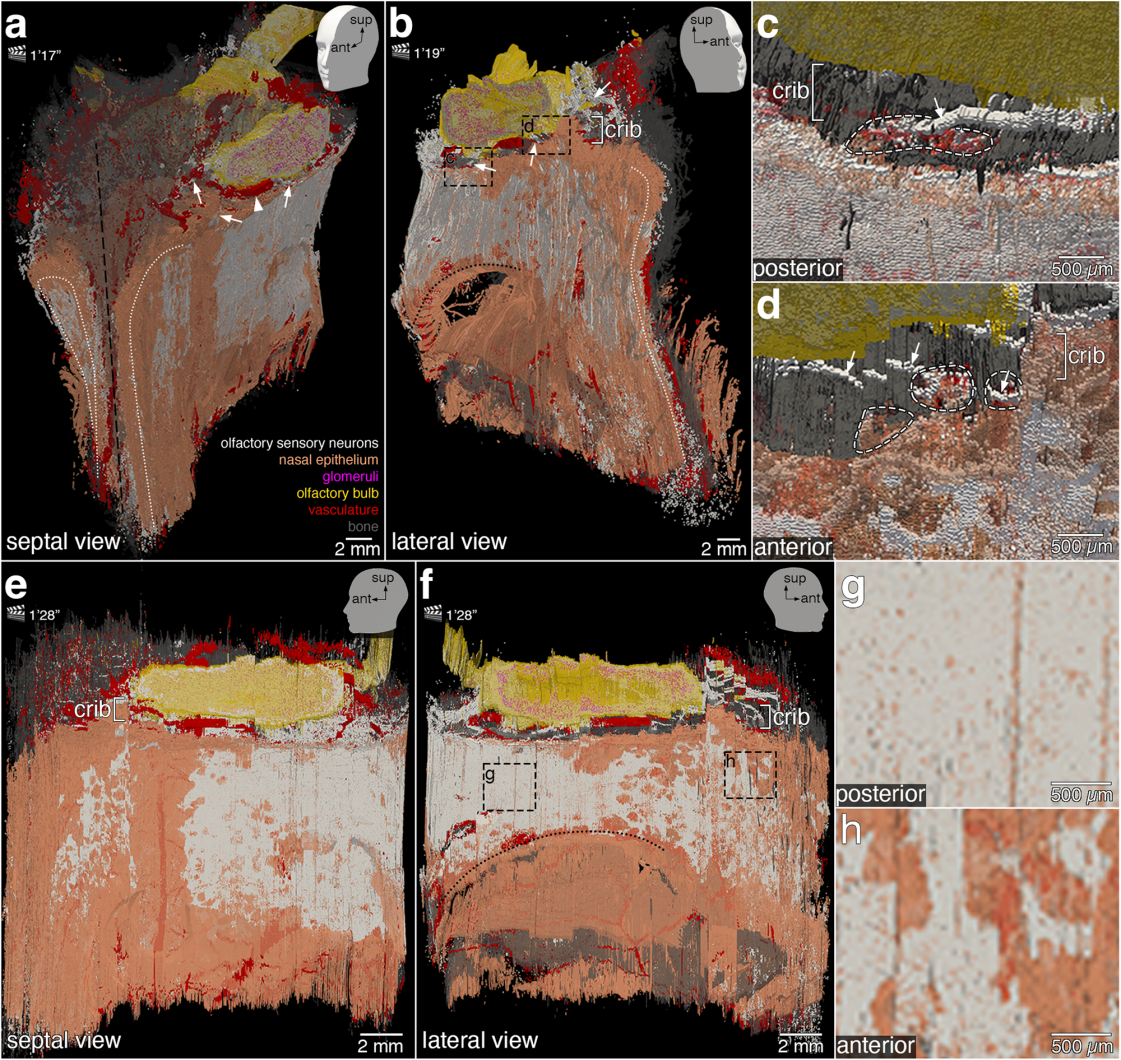
More still views of the 3D reconstruction. The head models with positional indicators of anterior (ant), lateral (lat), and superior (sup) in the top right corners indicate the angle of the still views on the 3D construction. The corresponding time points in Supplementary Movie 1 are indicated next to the clapperboards, as minutes and seconds. Images were taken in ParaView. Slicing the 3D reconstruction virtually along a parasagittal plane through the left nasal cavity exposes the nasal epithelium (orange) lining the septal aspect (**a**) and the lateral aspect (**b**) of the left nasal cavity. The distribution of OSNs (white) is irregular and the borders of the area occupied by OSNs are serrated. At the anterior face of the specimen, the luminal surface of the nasal epithelium of the left and right nasal cavities (white dotted lines) was physically cut. The curvature of the superior turbinate (black dotted line; **b**) is visible in the posterior aspect above a hole in the specimen. The perpendicular plate of the ethmoid bone (septum, black dashed line; **a**) lies in a midsaggital plane. Fila olfactoria (arrows) traverse the cribriform plate (crib). The accessory olfactory artery (arrowhead) is partially visible. High-magnification views of the dashed rectangles in (**b**) show posterior fila olfactoria (**c**) and anterior fila olfactoria (**d**) (arrows) traversing the cribriform plate through foramina (white dashed lines) toward the olfactory bulb (yellow). Parasagittal still views of the septal aspect (**e**) and lateral aspect (**f**) of the left nasal cavity highlight the irregular topography of OSNs within the nasal epithelium. In the lateral aspect (**f**), OSNs do not extend below the curvature of the superior turbinate (black dotted line). High-magnification views of the dashed rectangles in (**f**) illustrate the difference in OSN density posteriorly (**g**, dense) compared to anteriorly (**h**, less dense).

**Fig. 8:**
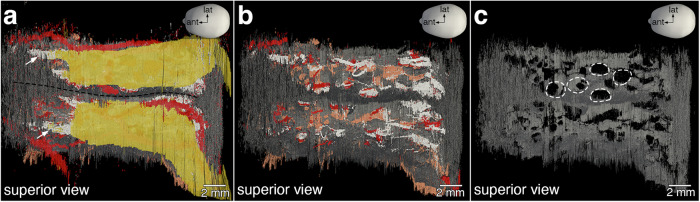
Foramina in the cribriform plate. Superior views. **a** The olfactory bulbs are separated anteriorly by the crista galli and posteriorly by the perpendicular plate of the ethmoid bone (black dashed line). Anteriorly, a few fila olfactoria (arrows) are visible. **b** A virtual dissection of the olfactory bulbs exposes multiple fila olfactoria traversing the cribriform plate and a network of arterial vasculature (red). **c** Isolating the cribriform plate (gray) exposes the foramina (white dashed lines). There are 17 foramina on either side of the specimen, for a total of 34 foramina.

The 3D visualization offers several didactic opportunities of interactive exploration and virtual manipulation (Supplementary Movie 1). Next, still views are shown for tilting the 3D reconstruction to observe it from an oblique angle, and cutting a slice out of the 3D reconstruction (Fig. 6); slicing the 3D reconstruction in freestyle and opening it (Fig. 7); and microdissecting the 3D reconstruction akin to physically dissecting a cadaver (Fig. 8).

### Gross anatomical organization, vasculature, and trajectory of OSN axons

Figure 6 presents a selection of still views captured from Supplementary Movie 1, with the corresponding time points indicated next to the clapperboards in the figure panels.

Tilting the 3D reconstruction and viewing it from an oblique angle gives a first impression of the gross anatomical organization (Fig. 6, top row). The left and right nasal cavities are lined with nasal epithelium and are separated by the nasal septum, which has the perpendicular plate of the ethmoid bone at its center (Fig. 6a). The cribriform (“sieve-formed”) plate of the ethmoid bone rests in the axial plane between the nasal and cranial cavities and the crista galli (“crest of the cock”) lies in the midsagittal plane between the olfactory bulbs. The dimensions of the olfactory bulbs are ~10 mm in the anterior-posterior dimension, ~2.4 mm superior-inferior, and ~3.5 mm medial-lateral. Posteriorly, the olfactory bulbs taper into a thin cylindrical shape—the beginning of the olfactory tract projecting to the cerebral cortex. Rotating the 3D reconstruction along a vertical axis, the dense vascularization of the lateral aspect of the left nasal cavity becomes apparent (Fig. 6b). Several arteries have been given names^14–16,58,59^.

Cutting out a 1 mm-thick virtual coronal slice illustrates how the OSN segmentation visualizes the trajectory of OSN axons across their entire length of several cm (Fig. 6, bottom row). On the lateral aspect, the olfactory epithelium does not extend inferiorly to the superior turbinate, but on the septal aspect, it extends much more inferiorly (Fig. 6c). OSN axons group together into olfactory axon fascicles ultimately forming fila olfactoria that traverse the cribriform plate ipsilaterally and innervate the glomerular array of the ipsilateral olfactory bulb (Fig. 6d).

### Olfactory epithelium, fila olfactoria, and foramina

Figure 7 presents another selection of still views captured from Supplementary Movie 1. The irregular and discontinuous topography of the olfactory epithelium can be readily appreciated by slicing the 3D reconstruction along a parasagittal plane in the left nasal cavity and viewing the two resulting parts from an oblique angle (Fig. 7, top row) and then opening it completely and viewing the two parts sideways (Fig. 7, bottom row). A still view of the right part of the parasagitally sliced 3D reconstruction reveals two broad areas of olfactory epithelium on the septal aspect of the left nasal cavity, separated by a superior-inferior running band of nasal epithelium devoid of OSNs (Fig. 7a). A still view of the left part of the parasagitally sliced 3D reconstruction displays the serrated contours of the olfactory epithelium on the lateral aspect (Fig. 7b). A thick filum olfactorium is seen innervating the left olfactory bulb posteriorly, close to an artery (Fig. 7c). Several fila olfactoria can be discerned anteriorly (Fig. 7d). In the 3D reconstruction, we manually counted 10 fila olfactoria laterally and 7 medially on the left side, and 9 laterally and 8 medially on the right side—34 in total. Opening the parasagitally sliced 3D construction completely, akin to opening a book and flattening the two adjacent pages, gives still views of the septal aspect (Fig. 7e) and the lateral aspect (Fig. 7f) of the left nasal cavity. The olfactory epithelium is denser posteriorly (Fig. 7g) than anteriorly (Fig. 7h).

### Counting foramina in the cribriform plate

Figure 8 shows a virtual microdissection of the 3D reconstruction, akin to physically dissecting the specimen. A virtual microdissection conducted above the cribriform plate provides still views of the foramina of the cribriform plate. Anteriorly, medial and lateral fila olfactoria can be discerned coursing toward the olfactory bulbs (Fig. 8a). After removing the olfactory bulb segmentation, more fila olfactoria are unveiled, ascending through foramina of the cribriform plate, and a network of vasculature below the cribriform plate is intermittently observed through the foramina (Fig. 8b). Removing all other structures to isolate the cribriform plate from the surrounding bone reveals the foramina (Fig. 8c). Exploring the 3D reconstruction, we counted 17 foramina on each side—34 in total.

### Interleaved sampling

Having processed the complete dataset of 1234 sections, we can evaluate the outcome of using only a subset of sections at a given sampling rate, which is called “interleaved sampling”. Note that, since each section was segmented independently, the CNN segmentations themselves are not affected by the interleaved sampling.

Quantitatively, we evaluated the impact of interleaved sampling in terms of a percentage error—the absolute change in percentage from the surface area or volume (Supplementary Fig. 5). At sampling rate of 1 in 4, there was a minor error in the measures compared to the complete dataset. At 1 in 32, the various measures still have an error of less than 5%, except for the glomerular volume. Qualitatively, we evaluated the outcome of registering two subsets, at sampling rates of 1 in 4 and 1 in 16. We found that the degradation in the quality of the visualization is still acceptable at 1 in 4 (Supplementary Fig. 6).

### Public access via Neuroglancer

**Fig. 9:**
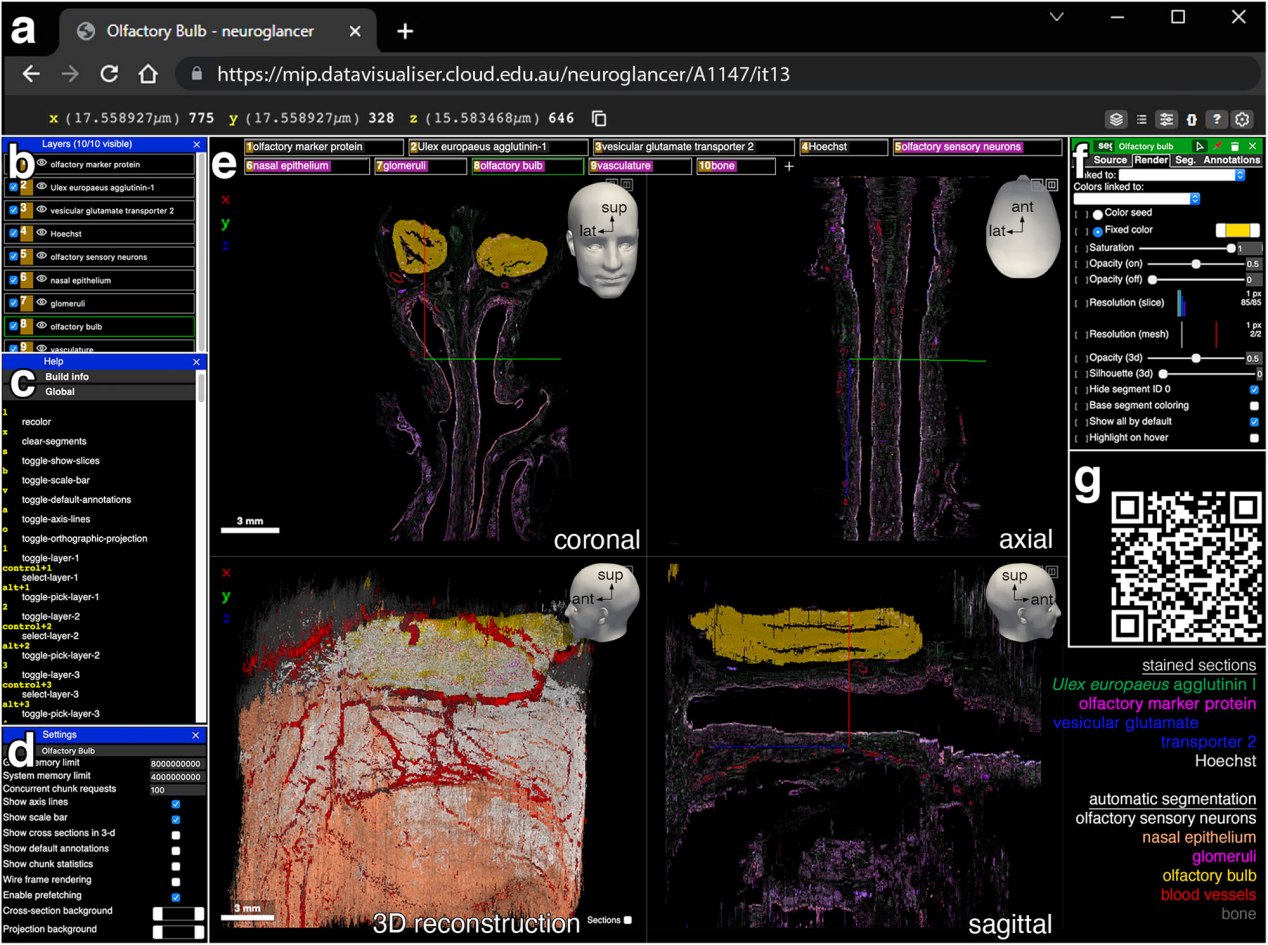
Visualization of the 3D reconstruction in the Neuroglancer platform. Neuroglancer is a WebGL-based, user-friendly viewer for 3D data. It hosts the scanned images and segmentation data of the complete dataset of 1234 sections of the olfactory *en-bloc* specimen extracted from case A1147. **a** The x, y, and z positions of the common origin of the colored axis lines in (**e**) are indicated at the left of the top menu bar. **b** The layers panel displays each of the fluorescence channels and CNN segmentations and controls the visibility of the channels and segmentations displayed in (**e**). **c** The help panel provides information on the keyboard shortcuts. **d** The settings panel enables the customization of the viewer. GPU and CPU memory usage can be adjusted. Helper tools such as axis lines and scalebar can be hidden from view. The background color can be changed. **e** The main visualization panel displays, by default, four views with the layers indicated along the top. The coronal slice (top left) displays the original orientation of the sections and segmentations. The axial slice (top right) and sagittal slice (bottom right) are generated by virtual reslicing of the 3D reconstruction. The complete 3D reconstruction (bottom left) can be rotated in 360°. Top right arrows indicate anterior (ant), lateral (lat), and superior (sup). Left-clicking the layers tabs toggles visibility and right-clicking activates the layer control (**f**) for that selection. Toggling the buttons in the upper right corner expands the view to a two-split or full-screen view. **f** The layer control panel allows for the manipulation of the displayed color, opacity, brightness, and contrast for each of the channels and segmentations. **g** QR code of the link to a compact (mobile-friendly) view of the model hosted on the Neuroglancer platform, without information (**b**–**d**) and (**f**) displayed. The link to the full view is https://mip.datavisualiser.cloud.edu.au/neuroglancer/A1147/it13.

The complete dataset, downsampled 16-fold (~5.8 GB per channel and ~1.45 GB per CNN segmentation, voxel volume 17.559 × 17.559 × 10 µm), is available online in a user-friendly format through Neuroglancer, a web-based platform (Fig. 9). The user can pan through 2D views in the original coronal plane, as well as in virtual axial and sagittal planes. The 3D visualization can be rotated in 360° and each channel and CNN segmentation can be viewed in customizable combinations.

### Computing the total number of olfactory sensory neurons

The OSN segmentation does not have single-OSN resolution and therefore does not allow us to directly obtain the total number of OSNs in the specimen. A complicating factor in counting OSNs in humans is that OSNs are distributed non-uniformly across the nasal epithelium.

**Fig. 10:**
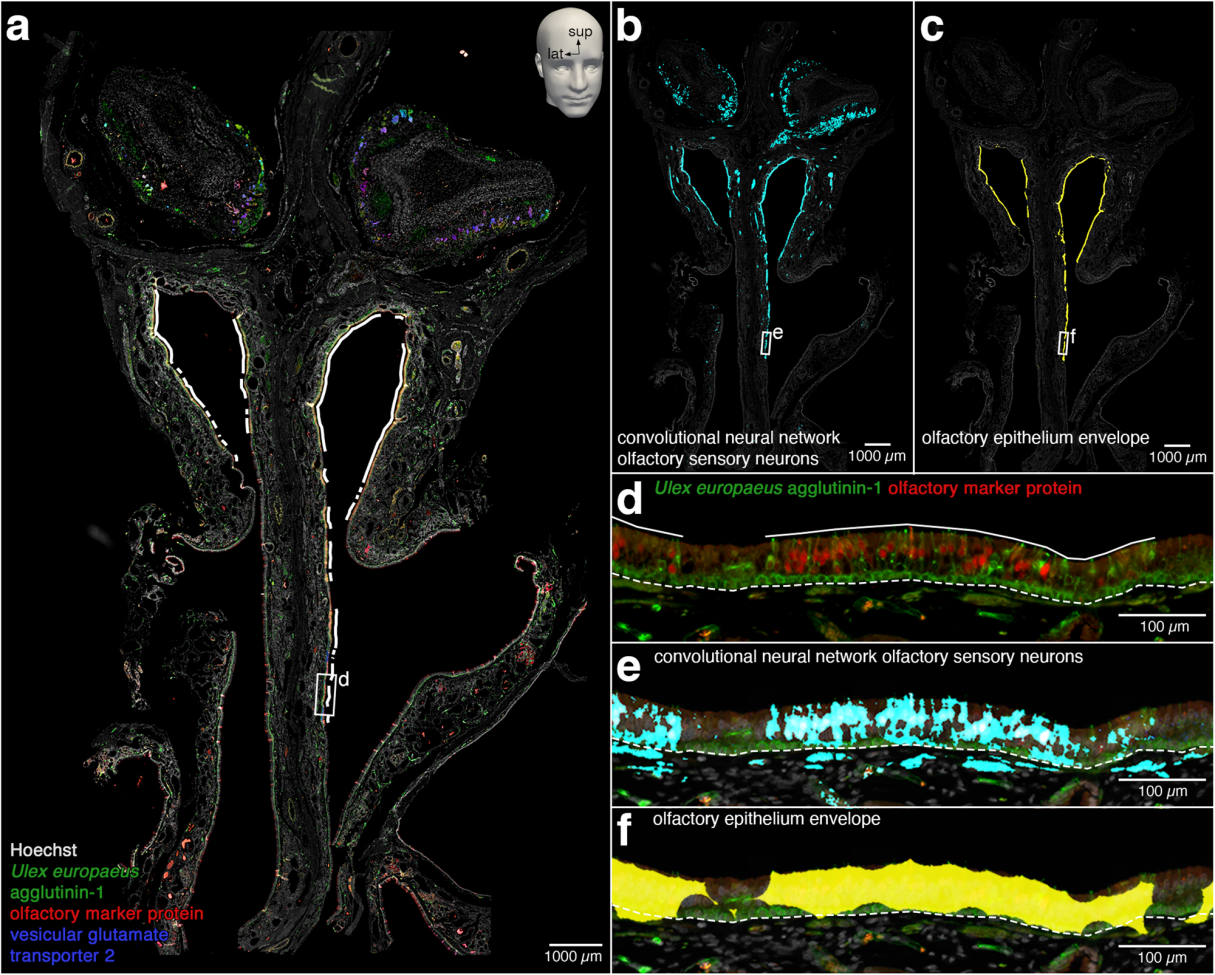
The envelope of the morphologically enclosed olfactory epithelium. **a** 10 μm coronal section (#1160) stained fluorescently for Hoechst (gray), *Ulex europaeus* agglutinin-1 (green), olfactory marker protein (red), and vesicular glutamate transporter 2 (blue). The interrupted white line represents the regions of the nasal epithelium that contain OSNs. **b** Merge of Hoechst with the CNN segmentation of OSNs (cyan). **c** Merge of Hoechst with the envelope of the olfactory epithelium (yellow). **d** High-magnification view of the rectangle demarcated in (**a**). Merge of *Ulex europaeus* agglutinin-1 (green) and olfactory marker protein (red) illustrates how OSNs in the nasal epithelium can be identified and counted manually. The solid white line indicates what was measured by the trained anatomist to determine the length of nasal epithelium that contains OSNs. The dashed white line indicates the basal lamina. **e** High-magnification view of the rectangle demarcated in (**b**). Merge with the CNN segmentation of OSNs (cyan). White represents overlap between the ground truth and CNN segmentations of OSNs and cyan represents overprediction by the CNN segmentation. The dashed white line indicates the basal lamina. **f** High-magnification view of the rectangle demarcated in (**c**). Merge with the CNN segmentation of OSNs within the nasal epithelium, morphologically closed along the apical-basal dimension by erosion and dilation, reveals the envelope of the olfactory epithelium (yellow) that was used to calculate the volume of the entire olfactory epithelium. The semi-circular “bites” in the contours of the envelope reflect the morphological operation of erosion using a disk of a diameter of 80 µm as a structuring element.

We computed the total number of OSNs in the specimen based on the concept of an envelope of the olfactory epithelium, in five steps (Fig. 10). First, using Fiji^60^, we counted the average number of OSNs per unit length (mm) of olfactory epithelium as 78.31 ± 4.65 (mean ± SEM), with an Abercrombie correction for overcounting, from manually counting 14,570 OSNs in 7 sections throughout the specimen. Second, further using Fiji, we calculated the average thickness of the olfactory epithelium as 67.23 ± 0.90 µm (mean ± SEM), from 240 measurements at 20 locations where OSNs were present, in 12 sections throughout the specimen. Third, with a section thickness of 10 µm, we computed the OSN density as 116,472.64 ± 7118.24 (mean ± SEM) per mm^3^ of olfactory epithelium, with the SEM calculated using Fieller’s theorem. Fourth, we calculated the volume of the olfactory epithelium as 22.97 mm^3^ from an envelope obtained by determining the intersection of the nasal epithelium segmentation and the OSN segmentation and closing the holes along the thickness of the epithelium with the morphological operations of erosion and dilation^61^. Fifth, extrapolating the OSN density per mm^3^ to the volume of the envelope of the olfactory epithelium yielded a computed total number of 2,675,323 ± 163,486 (mean ± SEM) OSNs. The 90% confidence interval from 2,406,390 to 2,944,259 was determined using the Delta method.

Taken together, by extrapolating manual counts of OSNs in a subset of sections to the calculated volume of the envelope of the entire olfactory epithelium, we computed a total of ~2.7 million OSNs in the specimen.

## Discussion

The *en-bloc* specimen of case A1147 measures ~1.92 cm in the anterior-posterior dimension, ~2.27 cm superior-inferior, and ~1.71 cm left-right, for a volume of ~7.45 cm^3^. A comparable *en-bloc* specimen from a 21-day-old mouse^62^ measures ~1 cm anterior-posterior, ~0.5 cm dorsal-ventral, and ~0.4 cm left-right, for a volume of 0.20 cm^3^. Thus, the dataset volumes contain ~37-fold more information in human compared to mouse. Manual segmentation and processing of the terabytes-sized dataset would have been prohibitively labor-intensive. Instead, we trained CNNs and made use of a high-performance computing infrastructure to process 1234 sections and to manage the computational complexity posed by the registration of this many images. Our computing approach efficiently manipulated the complete dataset in a distributed manner across multiple processors and within reasonable processing times. Our inter-block and intra-block multiresolution pyramid approach to the most computationally-expensive phase of the project—the registration—overcame some of the file size limitations while preserving the microanatomy in the resulting 3D reconstruction.

Consistent with the long history of research on the human *regio olfactoria*^21^, we observed a complex topography of the olfactory epithelium in the specimen. Some of the adjectives and prose that have appeared in the literature are: degenerated, eroded, heterogeneous, intermixed, interrupted, interspersed, non-continuous, non-uniform, patchy, porous, and serrated^18,19,21,30,63–70^ and an archipelago^71,72^. Studies on the fetal human olfactory epithelium are far and few between^45,64,69^, and, like in mice and rats, it presents as continuous and is outlined by a regular border with the respiratory epithelium. This pristine topography appears to break down already at 1–2 years of age in humans^65^. In future studies, markers can be employed to identify sustentacular cells, enabling the segmentation of so-called “aneuronal” olfactory epithelium: areas devoid of OSNs but still containing sustentacular cells and basal cells^30,66,69,73,74^.

The OSN segmentation that we report here does not have single-OSN resolution. We computed a total of 2,675,323 ± 163,486 (mean ± SEM) OSNs in the specimen. This computed number could be a lower estimate because some OSNs may not have been included in the specimen, particularly in the posterior aspect. Due to the idiosyncratic topography of the human olfactory epithelium and the non-uniform distribution of OSNs, the unknown sampling error made by analyzing small samples^75–78^ has precluded attempts in the literature at computing or even estimating the numbers of OSNs in a human being.

There is currently no computational approach to reliably delineate individual human glomeruli as units and count them^34^. The glomerulus segmentation that we report here ought to be seen as the segmentation of the glomerular component of the olfactory bulb. Nonetheless, the following quantitative considerations might be informative. A range of 2975 to 9325 glomeruli per human olfactory bulb was proposed in one study^33^. The olfactory bulb with the largest number of glomeruli in that study^33^ had a volume of 65.31 mm^3^, which is comparable to the volumes of 66.32 and 68.21 mm^3^ that we measured in the specimen. Adopting their theoretical reasoning^33^ that human glomeruli are spheres with an average diameter of 59.60 µm, our estimates for the specimen are 13,983 glomeruli left and 12,088 right, based on glomerular volumes of 1.55 mm^3^ left and 1.34 mm^3^ right. At an average diameter of 55.15 µm (their lower end^33^), our estimates are 17,648 glomeruli left and 15,257 right; and at an average diameter of 65.13 µm (their higher end^33^), our estimates are 10,715 glomeruli left and 9263 right. Of note, small differences in the hypothetical average diameter of glomeruli viewed as spheres result in large differences in the estimates of the numbers of glomeruli, due to the third power of the radius in the formula for the volume of a sphere.

When our computed count of 2,675,323 OSNs total is divided by our estimate of 26,071 glomeruli total (19,978 at the higher end of average glomerular diameters and 32,905 at the lower end), there would be an average of ~103 OSNs innervating a glomerulus (~134 and ~81 at the higher and lower ends, respectively), provided that OSN axons do not branch. In 21-day-old mice, we obtained an estimate of ~6,600,000 OSNs^79^, and with ~3600 glomeruli in a mouse olfactory bulb^42^, that amounts to ~917 OSNs per glomerulus. Puzzlingly, there may thus be a difference of an order of magnitude between humans and mice in terms of the average number of OSNs per glomerulus. This major quantitative difference may well translate into major qualitative differences in the coding logic of olfactory sensory processing between humans and mice. In this regard, it remains entirely unclear, and experimentally difficult to determine, whether a human glomerulus is innervated homogeneously by axons of OSNs expressing the same odorant receptor gene, as is the case in mice.

On the other hand, the subpopulations of OSNs expressing a given odorant receptor gene may turn out to be surprisingly similar in humans and mice. With 2,675,323 OSNs in the specimen and 389 odorant receptor genes in the human genome^43^, there would be an average of 6877 OSNs per odorant receptor gene. This number is comparable to the 5784 OSNs per odorant receptor gene in mice: ~6,600,000 OSNs divided by 1141 odorant receptor genes in the mouse genome^43^.

Going forward, interleaved sampling will reduce the time and resources for the computationally-expensive phase of registration and will increase the throughput of the workflow. An appreciation of the variability among human subjects with normal olfactory function would provide a baseline and framework for a quantitative approach to the microanatomical aspects of the pathobiology of olfactory dysfunction in the two most common neurodegenerative diseases, Parkinson’s and Alzheimer’s disease, as well as of olfactory dysfunction that persists after a SARS-CoV-2 infection. By using other panels of antibodies or adding fluorescence channels, CNNs could be trained to distinguish between mature and immature OSNs; to identify sustentacular cells and ciliated respiratory cells; to segment lymphatic vessels; and to detect pathological accumulations like α-synuclein (Parkinson’s) and phosphorylated tau (Alzheimer’s). A more distant objective would be to assemble similar 3D reconstructions of the entire human brain. At an interleaved sampling rate of 1 in 4 and with 4 fluorescence channels per subset (Hoechst being a shared channel), a palette of 13 fluorescence markers would segment myriad structures of interest.

An obvious limitation of our study is that it is an n of 1. We do not know how representative case A1147 is for the human species. Another related, limitation is that the throughput is ultra-low. Our objective was to provide a proof of principle for how to study histologically the microanatomy of the human olfactory projection; this study ought to be viewed as a case report. The specimen was from an aged individual, but most individuals who bequeath their bodies for research are aged. Due to the physical size of the scanned images and the computational demands required to handle the dataset, we downscaled the images to a 1:4 resolution. With more computational power and computational advances, a higher resolution will be attainable. Our methodology^80^ was developed for the segmentation, registration, and reconstruction of a specific specimen and should not be understood as a general methodology for such specimens. Demonstrating generalizability requires assessing how well the workflow performs with other specimens and may require additional training and modifications. Last but not least, the most labor-intensive phase in terms of hands-on time was the ground truth segmentation by a trained anatomist. This time-consuming phase precludes the creation of a veritable pipeline and upscaling the sample size by, say, an order of magnitude. Novel approaches to generating a virtual ground truth must be explored.

## Methods

### Cadavers and specimens

Postmortem *en-bloc* specimens were collected at the Human Anatomy Lab of The University of Auckland through the Human Body Bequest Programme, which is governed by the New Zealand Police, and in compliance with the Human Tissue Act 2008 from the New Zealand Parliament. Full written informed consent was obtained from the family prior to any procedure taking place. All ethical regulations relevant to human research participants were followed. The donors had no documented history of olfactory dysfunction and died in the prepandemic era. Upon arrival at the Human Anatomy Lab, cadavers were embalmed via the common carotid and femoral arteries, flushing first with 5 liters of saline solution followed by ~20 liters of Dodge Anatomical Arterial Michigan Mixture. The brain was removed from the cadaver in such a way that the olfactory bulbs remained attached to the cribriform plate of the ethmoid bone. Incisions were made posterior to the frontal sinus, anterior to the sphenoid bone, and lateral to the olfactory bulbs, and a specimen containing the olfactory projection and ancillary structures was extracted *en bloc*. The specimens were further fixed with 15% formalin in 0.1 M phosphate buffer for 24 h at room temperature and decalcified with 0.45 M EDTA in phosphate-buffered saline (PBS) for 3 months at room temperature. The EDTA buffer was changed every 4–6 weeks and the progress of decalcification was manually checked for completion. The decalcified specimens were trimmed of excess tissue, dehydrated in a graded ethanol series, cleared, and infiltrated with molten paraffin wax. The paraffin blocks were cooled to 4 °C and cut serially into 10 µm coronal sections on a rotary microtome (Leica Biosystems, Cat#RM2235), from the anterior to the posterior face of the specimen. Sections were floated in a water bath (Leica Biosystems, Cat#HI1210) at 38–40 °C, mounted on UberfrostPlus slides (InstrumeC, Cat#VS5116W20F-PLUS), air-dried, and stored at room temperature. A grand total of 11,805 sections were cut: A1130, *n* = 1500 sections (two thirds of the specimen sectioned); A1144, *n* = 2097 (sectioned to completion); A1147, *n* = 1923 (to completion); A1161, *n* = 1953 (to completion); A1164, *n* = 2100 (to completion); A1177, *n* = 2232 (to completion).

### Chromogenic staining of sections for case selection

To determine the preservation and tissue quality of the specimens, sections were sampled at an interval of 1500 µm (i.e., every 150th section) and stained chromogenically. Slides were dewaxed by heating to 60 °C for 1 h, immersed 2 × 20 min in xylene, rehydrated in an ethanol series, followed by heat-induced antigen retrieval using Tris-EDTA with Tween buffer (pH 9.0) heated to 121 °C and left to cool for 2 h in a pressure cooker (Aptum Biologics Ltd., Cat#2100 Antigen Retriever). Slides were washed for 10 min in PBS and endogenous peroxidases were blocked with 50% methanol/1% H_2_O_2_ for 20 min at room temperature. Slides were washed 3 ×10 min in PBS, blocked for 1 h at room temperature in PBS with 10% normal donkey serum (Genetex, Cat#GTX73245), then incubated for 72 h at 4 °C with goat anti-OMP antibody (1:10,000; FUJIFILM Wako Shibayagi, Cat#019-22291-WAKO) diluted in PBS with 1% normal donkey serum and 0.2% Triton. Slides were washed 3 × 10 min in PBS and incubated for 2 h at room temperature with biotinylated donkey anti-goat secondary antibody (1:1,000, Jackson ImmunoResearch Laboratories, Cat#705-065-003) diluted in PBS with 1% normal donkey serum and 0.2% Triton. Slides were washed 3 ×10 min in PBS before and after incubation in ExtrAvidin-Peroxidase (1:1000; Sigma, Cat# E2886-1ML) for 1 h at room temperature. Visualization was carried out with 3, 3-diaminobenzidine (DAB) chromogen (Sigma-Aldrich, Cat#D5637) for 20 min at room temperature. Slides were washed with PBS, then with water. Slides were counterstained with Alcian blue (for mucins) and Hematoxylin (for nuclei), as follows: after a 5 min wash in water, slides were incubated in Alcian blue (1% in 3% acetic acid, pH 2.5) for 10 min, then with Hematoxylin (Gill II, Leica Biosystems, Cat#3801520) for 1 min, differentiated with 1% acid alcohol, blued in 1% lithium carbonate, washed in water, dehydrated in an ethanol series followed by 3 ×30 min Xylene baths. Slides were coverslipped using the non-aqueous mounting medium DPX new (Sigma-Aldrich, Cat#1.00579). These chromogenically stained lines were inspected under a Nikon Eclipse NiE microscope with a motorized stage, and tiled scans were captured using NIS elements software and a Nikon DS-Ri2 brightfield camera.

### Case A1147

The *en-bloc* specimen extracted from case A1147 had the most intact morphological appearance among the six available specimens and was selected for the 3D reconstruction. The medical history of this 88-year-old male includes type 2 diabetes mellitus, hyperlipidemia, ischemic heart disease, and two-stroke events, but no documented history of olfactory dysfunction. Apathetic-type dementia was diagnosed three years before death and progressed to severe dementia in the week before death. The cause of death was sepsis and the postmortem delay was ~24 h. The *en bloc*-specimen measured after trimming ~1.92 cm in the anterior-posterior dimension, ~2.27 cm superior-inferior, and ~1.71 cm left-right, corresponding to a volume of ~7.45 cm^3^. This cadaveric tissue sample is referred to as “the specimen”.

### Fluorescence staining of sections of the specimen

A subset of 72 sections/24 slides of the original series of 1923 sections was used for validation, as follows: 36 sections/12 slides for chromogenic labeling, 6 sections/2 slides to check cilia quality, 6 sections/2 slides for RNAscope, and 24 sections/8 slides for testing fluorescent stainings. The remaining 1851 sections were subjected to quadruple fluorescence staining. Slides were dewaxed, rehydrated in an ethanol series, followed by heat-induced antigen retrieval using the antigen retrieval buffer Tris-EDTA with Tween (pH 9.0) heated to 121 °C and left to cool for 2 h in a pressure cooker. Slides were washed 3 ×10 min in PBS and blocked for 1 h at room temperature in 10% normal donkey serum. Slides were incubated for 72 h at 4 °C in a humidified chamber with primary antibodies goat anti-OMP (1:2000) and guinea pig anti-VGLUT2 (1:1000; Frontier Science, Cat#VGluT2-GP-Af670-1) together with biotinylated *Ulex europaeus* agglutinin-1 (UEA1) (1:2000; Vector Laboratories, Cat#B-1065), diluted in PBS with 1% normal donkey serum and 0.2% Triton. Slides were washed 3 ×10 min in PBS and then incubated for 2 h at room temperature with fluorescently conjugated secondary antibodies Alexa Fluor Plus 594 donkey anti-goat (1:500; ThermoFisher, Cat#A32758), Alexa Fluor 647 donkey anti-guinea pig (1:500; Jackson ImmunoResearch Laboratories, Cat#706-605-148), together with Alexa Fluor 488 streptavadin (1:500; ThermoFisher, Cat#S32354) and Hoechst 33342 (1:20,000; MolecularProbes, Cat#H1399), diluted in PBS with 1% normal donkey serum and 0.2% Triton. Slides were washed 3 ×10 min in PBS, coverslipped with antifade mountant ProLong Diamond (ThermoFisher, Cat#P36961), sealed around the edges using nail polish, and stored at 4 °C.

### Scanning of sections of the specimen

The 1851 sections/617 slides were scanned as a three-level z-stack using a PANNORAMIC MIDI II fluorescence digital slide scanner (3DHISTECH) with a 20× objective (0.8 NA) and a resolution of 0.274 µm per pixel. Filter sets were: DAPI (F36-500), 377/50 nm Ex (excitation) and 447/60 nm Em (emission); EGFP (F36-526), 482/18 nm Ex and 520/28 nm Em; TxRed/mCherry/Alexa594 (F26-310), 560/40 nm Ex and 635/60 nm Em; and Cy5 (F46-006, AHF), 620/60 nm Ex and 700/75 nm Em. Files were saved as JPEGEX, 12 bit, with 80% quality, taking ~15 GB per slide and ~12.2 TB in total. The three sections on a slide were annotated with the slide number and A, B, C in CaseViewer (3DHISTECH). The extended focus (merged z-stack) of each of the four channels of each section was exported. The BigTIFF, 1:4 resolution (1.097 µm per pixel), grayscale, and lossless compression (~200 MB per channel per section) files were padded to 20,736  × 15,616 pixels so that all images had the same dimensions. These files were renamed with sequential identifiers 0001-1923. The padded 1:4 resolution is henceforth referred to as “the original resolution”.

Of the original series of 1923 sections, 689 sections (35.8%) were not incorporated in the complete dataset, for one of three reasons. First, 72 sections were used for validation, as explained above, leaving 1851 sections for fluorescence staining and scanning. Second, 32 of these 1851 sections (1.7%) did not image properly during the scanning. Third, the poor quality of 585 of these 1851 sections (31.6%) would have impaired the automatic segmentation and registration protocols. Of these 585 sections, over 70% were excluded due to tissue damage (such as excessive deformation, sectioning damage, and missing pieces), and the remainder were largely excluded due to tissue folding over itself or the olfactory bulb sections becoming detached (Supplementary Fig. 2). Most of the non-included 689 sections were from the posterior (40%) and anterior (28%) quarters of the specimen. The largest sets of consecutively non-included sections consist of 16, 13, 11, 10, 10, 10, and 10 sections (total of 80 sections), representing 12% of the 689 sections that were not included. Most sets of consecutively non-included sections consist of ≤5 sections (total of 467 sections), representing 68% of the 689 sections that were not included; of these, 191 non-included sections were solitary in that the preceding (anterior) and following (posterior) sections were included. The remaining 142 sections (20%) are from sets of consecutively non-included sections numbering between 6 and 9. The final number of included sections is 1234, representing 64.17% of the original series of 1923 sections. These 1234 sections are referred to as “the complete dataset”.

### Manual segmentation to generate the ground truth

Six structures of interest were manually segmented into four sets of six sequential sections that evenly sampled the specimen. These 24 sections served to generate a reference dataset that formed the ground truth for training the CNNs for automatic segmentation; 20 sections formed the training dataset and 4 sections formed the validation dataset. After initial training, up to 31 additional sections were segmented manually to further train and validate the CNNs. Finally, two sections (#850 and #1401) were manually segmented and served as testing sections to test the performance of the CNN after the phase of training and validation (Supplementary Fig. 3).

The six structures of interest were manually segmented using Amira software (FEI, Hillsboro, OR, USA) on a Precision 7920 tower workstation (DELL) with a Wacom Intuos Pro Medium tablet. Each structure of interest was identified using the information available from all four stains: Hoechst, UEA1, OMP, and VGLUT2. The structures of interest are nasal epithelium, OSNs, glomeruli, olfactory bulbs, vasculature (arterial), and bone. For the segmentation of the nasal epithelium, no distinction was made between respiratory and olfactory epithelium. To manage display lag due to the large file sizes after the images were loaded into Amira, sections were sliced into six regions of interest that were extracted and segmented independently of each other. Afterwards they were merged again (resampled) to form a complete section.

In each section, all structures of interest were segmented using a combination of thresholding and manual tracing. Interactive thresholding was used to optimally display the structures of interest. A label analysis was then applied with an analysis filter and a mask was generated using threshold-by-criterion to filter objects too large or too small to be the structure of interest. Additionally, using the arithmetic computation, values from channels were added and subtracted from each other to offer alternative visualizations of specific structures of interest. In the segmentation editor, each structure of interest was given a unique label and the tools of masking, brush, lasso, and magic wand, were used together with threshold tools to complete the segmentation of a structure of interest.

### Automatic CNN segmentation

A 2D U-Net architecture was adjusted for the task to achieve a good trade-off between manual segmentation effort and segmentation accuracy (Supplementary Fig. 3). As the task of manual annotation is labor-intensive (up to 34 h per section) and demands the sustained attention of a trained anatomist, the number of ground truth sections, and therefore the size of the dataset, needed to be kept relatively small. To achieve high-accuracy training with such a relatively small dataset, two strategies were followed: (i) a bootstrap approach to actively check the network accuracy for each new batch of manually segmented sections, and then add more sections if the accuracy remained low; and (ii) shallower architectures to reduce the degrees of freedom to be trained. Strategy (ii) favored the use of a 2D U-Net with a minimal number of features at each resolution, resulting in the architecture presented below.

The ground truth dataset of 24 sections was divided into a training and validation split of 20 and 4. For each of the six structures of interest, an independent CNN was trained using TensorFlow (v2) on Python 3. The CNNs chosen for this study are based on the U-Net architecture^44^. A bootstrap approach was employed starting with the initial training dataset and where the trained CNN could not achieve the desired DSC, the lower-quality sections in the complete dataset were manually corrected and added to the ground truth. The corrected sections together with the original dataset of 24 sections were then used to further train the CNN for the structure of interest. This process was repeated until the desired DSC was achieved or there were diminishing returns.

The CNNs take an input tensor of size 512 × 512 × 4, representing a 512 pixels width by 512 pixels height region of the section, and by four channels (Hoechst, UEA1, OMP, VGLUT2). During training, the sections were cropped randomly to allow for an even sampling of the entire section with overlaps so that only the center pixels (which contain more information for the CNN to make decisions on) were used for segmentation.

A 3 × 3 convolution kernel size was employed to extract local features and a binary cross-entropy loss function was utilized to measure the discrepancy between predicted and ground truth segmentations. The ADAM optimizer in the TensorFlow framework was chosen to expedite the convergence of the model, with a batch size of 32.

The architecture comprises an encoder, a bottleneck, a decoder, and an output layer. The bottleneck connects the outputs of the encoder to the inputs of the decoder and, in turn, the output layer post-processes the outputs of the decoder. At the same time, the encoder has skip connections to the decoder as proposed in the original U-Net architecture^44^ (see the concatenation operators in the description below). The encoder, bottleneck, decoder output layer architectures are as follows:

· Encoder layer 1: Block composed of two 2D convolution operators with a 3 × 3 kernel extracting 8 features each, followed by a batch normalization and a 2 × 2 max pooling operations.

· Encoder layer 2: Block composed of two 2D convolution operators with a 3 × 3 kernel extracting 16 features each, followed by a batch normalization and a 2 × 2 max pooling operations.

· Encoder layer 3: Block composed of two 2D convolution operators with a 3 × 3 kernel extracting 32 features each, followed by a batch normalization and a 2 × 2 max pooling operations.

· Bottleneck: Block composed of two 2D convolution operators with a 3 × 3 kernel extracting 64 features each, followed by a batch normalization and a 2D transpose convolution operator with a 2 × 2 kernel.

· Decoder layer 3: Block composed of a concatenation operator fusing the Encoder layer 3 (the before max pooling operation) and the Bottleneck outputs, followed by two 2D convolution operators with a 3 × 3 kernel extracting 32 features each, a batch normalization operator and a 2D transpose convolution operator with a 2 × 2 kernel.

· Decoder layer 2: Block composed of a concatenation operator fusing the Encoder layer 2 (the before max pooling operation) and the Decoder layer 3 outputs, followed by two 2D convolution operators with a 3 × 3 kernel extracting 16 features each, a batch normalization operator and a 2D transpose convolution operator with a 2 × 2 kernel.

· Decoder layer 1: Block composed of a concatenation operator fusing the Encoder layer 2 (the before max pooling operation) and the Decoder layer 3 outputs, followed by two 2D convolution operators with a 3 × 3 kernel extracting 8 features each and a batch normalization operator.

· Output layer: Block composed of a 2D convolution operator with a 3 × 3 kernel extracting 2 features followed by softmax activation yielding the class probability of each pixel.

All images were utilized in their original resolution except for the olfactory bulb, which was downsampled fourfold (4.390 µm per pixel). Because the olfactory bulb is one order of magnitude larger than the other structures of interest, only its large-scale features are useful in the recognition tasks, and for the training of this CNN, the downsampled resolution gave faster and more accurate results than the original resolution. All 2D convolution operators use rectified linear unit (ReLU) as activation functions.

To reduce the experimental variation effect resulting from the staining and imaging process, an intensity augmentation was performed by randomly raising or lowering the channel intensities at each training step. We draw values from a uniform distribution of [−0.01, +0.01] to add to each individual channel during training. With the maximum intensity being 1, the distribution is informed by the intensity variation range of the images. The intensity was only augmented for less than how much it varied across the complete dataset. The objective was to force the CNN to learn that only the relative intensity between the neighboring pixels affects the segmentation results and that the overall intensity of a channel is not relevant. The CNN could then make consistent predictions across the complete dataset despite variations in channel brightness.

We trained the CNN for ~100 epochs for five structures of interest and ~400 epochs for the olfactory bulb. Preliminary training runs enabled an estimate of the number of epochs it would take for the CNN to be trained. By the end of the training, the binary cross-entropy on the validation dataset had stopped improving for at least 10 epochs. The 100 epochs were divided into three stages with an initial learning rate of 1.0E-04 (large), decreased gradually to 5.0E-05 (medium), and finally to 1.0E-05 (small). A CNN was trained for ~35 epochs at each learning rate. The CNN for the olfactory bulb was trained in two stages of 200 epochs, with large and small training rates. We decreased the learning rate so that the CNN could initially explore the solution space with a sufficiently large step size and, once a local minimum was found, converge to this minimum to achieve the optimal solution within the region. Note that this varying learning rate is different from the built-in adaptive step size in the ADAM optimization algorithm. At the end of each stage, the best-performing set of weights in the validation dataset was selected as the initial training model for the next stage with a smaller learning rate. To mitigate overfitting, a dropout rate of 0.2 was applied before each max-pooling operation in the encoder. With binary segmentation being performed, the CNN outputs a floating-point number between 0 and 1 for each pixel, which represents the probability for it to be a foreground or a background pixel, and this number is used to calculate the binary cross-entropy (the lower, the better).

A threshold value is needed to determine the cut-off between foreground and background. Due to the disproportional foreground vs. background pixel counts, the dataset is imbalanced. Therefore, the following metrics were analyzed to determine classification accuracy: Precision = true positives/(true positives + false positives), Recall = true positives/(true positives + false negatives), and DSC = (2 * Precision * Recall)/(Precision + Recall). The definition of DSC between two masks X and Y is

1 $${DSC}\left(X,Y\right)=\,\frac{2* {{|}}X\cap Y{{|}}}{\left|X\right|+{|Y|}}$$

where $\left|X\right|$ is the number of 1-valued pixels in a mask, and $X\cap Y$ is the intersection of 1-valued pixels between *X* and *Y*. In (1), *X* is ground truth segmentation and *Y* is CNN segmentation. Precision measures the quality of correct classification and Recall measures the quantity of correct classification. The DSC represents the intersection of the CNN segmentation and the manual segmentation divided by the union of the two and is a balance between Precision and Recall. We determined the classification threshold by choosing the cut-off value that gives the best DSC in the validation dataset. The threshold associated with the best-performing model was then used as the final threshold for that CNN.

The CNN segmentation and 3D reconstruction were assessed for aberrant and erroneous structures as well as continuity between sections by a trained anatomist. To test that the CNNs were performing as expected and were not overfitted to the training and validation datasets, a further two sections unseen by the CNNs were segmented by a trained anatomist, creating a second groundtruth dataset for testing the CNNs in an unbiased manner.

### Anatomy-based registration

Tissue deformations during the processes of fixation, sectioning, and staining result in global and local misalignments across sections. To register the individual sections and visualize the specimen in 3D, a two-step registration process was implemented. The first step (intra-block registration) registers fixed blocks of five sequential sections, and the second step (inter-block registration) interpolates the registration between adjacent blocks. In the intra-block registration, the complete dataset was divided into blocks of five sections and each block was registered independently. All blocks were submitted to a high-performance computing cluster and processed in parallel across 96 available Intel Xeon Gold 6136 CPUs. The inter-block registration process ensures that a smooth and more realistic transition of the anatomical structures through the axis of acquisition is obtained, coping with the so-called “banana effect”—a registration artifact that causes an artificial straightening of a curved object when reconstructing it using only cross-sectional information^54^.

For both steps, two consecutive registration operations were performed: an intensity-based registration using the four channels of the entire section for pre-registration, followed by a mask-based registration using only the vasculature and nasal epithelium segmentations (obtained from the CNNs) for a fine-grained registration. Vasculature and nasal epithelium were chosen because they have a relatively continuous structure across the specimen. The individual image registration operations were performed by using the open-source C++ SimpleElastix library^53^, which is built on top of the Insight Toolkit (https://itk.org). Our code uses the Python API of the library.

For each intra-block registration, the block reference section for registration was identified,$\,{\hat{X}}_{k}^{{{\rm{intra}}}}$, as the section with the highest DSC of vasculature and nasal epithelium with respect to the other sections in the block. To this end, a combined vasculature and nasal epithelium mask was defined as

$${X}_{i}^{c}={X}_{i}^{v}\vee {X}_{i}^{e}$$

where ${X}_{i}^{v}$ and ${X}_{i}^{e}$ are the masks for the vasculature and nasal epithelium for the *i*-th frame in the complete dataset and $\vee$ is the Boolean OR operator. Using the combined masks, the reference section was chosen as the one with the highest DSC agreement in the block, i.e., ${\hat{X}}_{k}^{{{\rm{intra}}}}={X}_{l}$ where

$$l=\,{{{\rm{argmax}}}}_{i\in {B}_{k}}{\sum}_{j\in \,{B}_{k}}{DSC}\left({X}_{i}^{c},{X}_{j}^{c}\right),\,{B}_{k}=\{j{\mathbb{\in }}{\mathbb{N}}{|}5(k-1)\le j < \, 5k\}$$

and ${B}_{k}$ is the set of the five section indices for the *k*-th block and DSC is defined by

$${DSC}\left(X,Y\right)=\,\frac{2* {{|}}X\cap Y{{|}}}{\left|X\right|+{{|}}Y{{|}}}$$

Using the reference section as the target, all the remaining sections in each block were registered using a multichannel block-matching strategy to align the four channels and six CNN segmentations of the sections. During this procedure, a rigid registration was performed followed by a non-rigid (B-Spline) registration with a multiresolution pyramid approach (16 and 13 levels for rigid and non-rigid respectively). Furthermore, random coordinate sampler, linear interpolator, mutual information, and gradient descent optimizer were used for selecting data points, interpolating intensity values, evaluating the alignment, and optimizing the transformation parameters, respectively.

This result was then used to initialize a mask-based registration for the block to fine-tune the registration based on the contours of the vasculature and nasal epithelium. In this process, we registered the combined vasculature and nasal epithelium masks (${X}_{i}^{c}$) instead of the channel intensities, using a non-rigid (B-Spline) registration with a three-level resolution pyramid and a random sparse mask sampler. The justification for only using three levels in the mask registration is to force the focusing on the smaller structures in the sections, otherwise, this mask-based step may undo the registration made by the previous intensity-based step.

At this point, we have locally registered each block in isolation and there will be an inconsistent registration between the block interfaces. To solve this issue, we proceeded to register each block using the pair of sections (one from each block) with the closest matching in terms of DSC. Specifically, we identified the pair ${\left({\hat{X}}_{{{\rm{fix}}}}^{{{\rm{inter}}}},{\hat{X}}_{{{\rm{mov}}}}^{{{\rm{inter}}}}\right)}_{k,k+1}$ as ${\hat{X}}_{{{\rm{fix}}}}^{{{\rm{inter}}}}={X}_{\hat{i}},\,{{\hat{X}}_{{{\rm{mov}}}}^{{{\rm{inter}}}}=X}_{\hat{j}}$ where

$$\hat{i},\hat{j}={{{\rm{argmax}}}}_{\left(i,j\right),i\in {B}_{k},j\in {B}_{k+1}}{DSC}\left({X}_{i}^{c},{X}_{j}^{c}\right)$$

${\hat{X}}_{{{\rm{fix}}}}^{{{\rm{inter}}}}$ is a section in the anterior block and ${\hat{X}}_{{{\rm{mov}}}}^{{{\rm{inter}}}}$ is a section in the posterior block. Then, we registered the pair ${\left({\hat{X}}_{{{\rm{fix}}}}^{{{\rm{inter}}}},{\hat{X}}_{{{\rm{mov}}}}^{{{\rm{inter}}}}\right)}_{k,k+1}$ using a rigid registration followed by a non-rigid (B-Spline) registration with a multiresolution pyramid approach (16 and 13 levels for rigid and non-rigid respectively). This registration yields the mapping ${\phi }_{k}(x)$ defined as the displacement at each pixel $x$ performed in section ${\hat{X}}_{{{\rm{mov}}}}^{{{\rm{inter}}}}$ to best register with ${\hat{X}}_{{{\rm{fix}}}}^{{{\rm{inter}}}}$, i.e., the registration done on the pixel $x$ in ${\hat{X}}_{{{\rm{mov}}}}^{{{\rm{inter}}}}$ is $x+{\phi }_{k}(x)$. Thus, we registered the N sections between ${\hat{X}}_{{{\rm{fix}}}}^{{{\rm{inter}}}}$ and ${\hat{X}}_{{{\rm{mov}}}}^{{{\rm{inter}}}}$ using the mappings ${\phi }_{k}^{1},{\phi }_{k}^{2},\ldots ,{\phi }_{k}^{N}$ defined as

$${\phi }_{k}^{i}(x)=\frac{i-1}{N}{\phi }_{k}(x)$$

As a result, we obtained a linear interpolation of the sections between ${\hat{X}}_{{{\rm{fix}}}}^{{{\rm{inter}}}}$ and ${\hat{X}}_{{{\rm{mov}}}}^{{{\rm{inter}}}}$, yielding a smooth registration at the interface between blocks k and k + 1. We refer to this process as the inter-block registration between blocks k and k + 1. We performed the inter-block registration on all blocks in sequential order, thereby obtaining a smooth registration of the complete dataset.

To determine the robustness of the anatomy-based registration when information from interleaved sampling is used, these processes were repeated using every 4th section and every 16th section of the complete dataset, representing ~16% and ~4% of the specimen.

### Visualization of the 3D reconstruction

The registered sections were visualized in Neuroglancer^57^, a web-based visualization interface for volumetric data that enables interaction with the images and 3D reconstruction. Prior to visualization, the channels and CNN segmentations were converted into Neuroglancer’s supported precomputed data format using the Python script available in their codebase. Additionally, the 3D surface meshes of the segmentation volumes were pre-generated in Neuroglancer’s mesh format with the marching cubes algorithm from the Python library of Neuroglancer. The image volumes were then divided into 3D blocks at multiple resolutions to enhance smooth and interactive cross-sectional visualization of 3D image data. Additionally, the open-source data analysis and visualization application ParaView^55,56^ was used to generate virtual slices and visualizations of the 3D reconstruction. ParaView was also used for the generation of a graphical animation shown in Supplementary Movie 1. The 3D head models in Figs. 1–3, Figs. 5–10, Supplementary Fig. 1, 4, 6, and Supplementary Movie 1 are from BodyParts3D, The database center for life science licensed under CC attribution-share alike 2.1 Japan.

### Volume calculations

Quantification of the volumes of OSNs, glomeruli, and olfactory bulbs was performed by counting the number of voxels of the structure of interest in the CNN segmentations. The sum of voxels across all 2D sections multiplied by the voxel volume (1.097 µm × 1.097 µm × 10 µm) gave the total volume of the structure of interest in 3D. The volume calculation in non-included sections was approximated as the linear interpolation of the nearest preceding (anterior) and following (posterior) included sections. In that manner, the complete dataset of 1234 sections was filled back to 1923 sections and quantifications could be made for the entire specimen.

### Septal-lateral and left-right divisions

In each section, the bone segmentation and the midpoint of the two olfactory bulbs were used as a guide to draw a spline to separate the left and right halves of the section and to define the left and right nasal cavities and left and right olfactory bulbs. Each nasal cavity was further split into the septal and lateral aspects by drawing a spline that traces points picked along the luminal side of the nasal epithelium on the septum and that cuts through the nasal epithelium in the olfactory cleft at the intersection with a line drawn from the center of the olfactory bulb.

### Computing the total number of OSNs in the specimen

First, a total of 14,570 OSNs were manually counted across seven sections that were randomly selected from the complete dataset using the cell counter plugin of Fiji ImageJ2 version 2.3.0/1.53n. An Abercrombie correction was applied to adjust for overcounting. We computed the average thickness of the olfactory epithelium by making 240 measurements at 20 locations where OSNs were present, in 12 sections throughout the specimen. Nucleus diameter, length, and thickness of olfactory epithelium were measured with the line tool in Fiji^60^. We determined the volume of the olfactory epithelium by expanding the OSN segmentation that intersected with the nasal epithelium segmentation to the full thickness of the nasal epithelium segmentation, closing the holes along the apical-basal dimension by applying the approach of morphological closing by erosion and dilation^61^ using as structuring element a disk with a diameter of 80 µm, which is similar to the thickness of the nasal epithelium. (Note that the envelope of the olfactory epithelium that is hereby obtained has a volume that is greater than the volume of the subcellular parts of the OSNs that are located within the olfactory epithelium.) To validate our automatic method of morphologically closing the olfactory epithelium (generating the “olfactory epithelium envelope”), we manually measured in 15 randomly selected sections the length of olfactory epithelium per section (adding up all the linear pieces where OSNs were present) and used a paired t-test to compare these manual measurements to the automatic measurements in the corresponding sections (*P* ≥ 0.83, not significant). The surface area of the olfactory epithelium was calculated by dividing the volume of the olfactory epithelium envelope by the average thickness of the olfactory epithelium and the SEM was estimated from the SEM of the thickness using the Delta method.

### Quantification with interleaved sampling

In a 10 µm tissue section, each 1.1 µm × 1.1 µm pixel in a single 2D CNN segmentation also contains volume information, resulting in a voxel of 1.097 µm × 1.097 µm × 10 µm. The voxel volume was adjusted accordingly to accommodate non-included sections. Quantification was carried out using the CNN segmentations with the results presented as length, area, and volume. To determine the error that would have been introduced by a reduced sampling rate of the specimen, quantifications were repeated using interleaved datasets that contained every 4th, every 16th and every 32nd section of the complete dataset (representing ~16%, ~4%, and ~2% of the specimen) and the errors are presented as a percentage difference from the results generated using the complete dataset (~64% of the specimen).

### Statistics and reproducibility

Comparisons between the total volume of the six structures of interest and the interleaved volumes are presented as a percentage change from the complete dataset volume. For the comparisons between the segmentations of the trained anatomist and the CNN, the values of DSC and binary cross-entropy of the validation dataset are presented as the mean ± SEM. The comparison between the trained anatomist’s measurement of the epithelium length and the calculated epithelium length, combining the measurement of the epithelium thickness (mean ± SEM) and the volume of the olfactory epithelium envelope (absolute number), was made using a paired t-test. OSN density was determined by dividing the manual cell counts of OSNs per unit length of olfactory epithelium (mean ± SEM) by the average thickness of the olfactory epithelium (mean ± SEM). OSN density is presented as mean ± SEM, with the SEM calculated using Fieller’s theorem. The Delta method was used to calculate the SEM and 90% confidence interval for the total number of OSNs in the specimen using the OSN density (mean ± SEM) and the volume of the olfactory epithelium envelope (absolute number).

### Reporting summary

Further information on research design is available in the Nature Portfolio Reporting Summary linked to this article.

## Supplementary information

Supplementary Information

Description of Additional Supplementary Files

Supplementary Movie 1

Reporting Summary

## Data Availability

The complete dataset of the *en-bloc* specimen of case A1147 is available online in a user-friendly format through Neuroglancer^57^, a web-based platform, at https://mip.datavisualiser.cloud.edu.au/neuroglancer/A1147/it13.

## Code availability

All original code has been deposited at Zenodo^80^ and is publicly available, at 10.5281/zenodo.7865523.

## References

[1] Killingley, B. et al. Safety, tolerability and viral kinetics during SARS CoV-2 human challenge in young adults. *Nat. Med.* **28**, 1031–1041 (2022). 35361992 10.1038/s41591-022-01780-9

[2] Boscolo-Rizzo, P. et al. Psychophysical assessment of olfactory and gustatory function in post-mild COVID-19 patients: a matched case-control study with 2-year follow-up. *Int. Forum Allergy Rhinol.* **13**, 1864–1875 (2023). 36852674 10.1002/alr.23148

[3] Doty, R. L., Deems, D. A. & Stellar, S. Olfactory dysfunction in parkinsonism: a general deficit unrelated to neurologic signs, disease stage, or disease duration. *Neurology* **38**, 1237–1244 (1988). 3399075 10.1212/wnl.38.8.1237

[4] Haehner, A., Hummel, T. & Reichmann, H. Olfactory loss in Parkinson’s disease. *Parkinsons Dis.* **2011**, 450939 (2011). 21687752 10.4061/2011/450939 PMC3109349

[5] Doty, R. L., Reyes, P. F. & Gregor, T. Presence of both odor identification and detection deficits in Alzheimer’s disease. *Brain Res. Bull.* **18**, 597–600 (1987). 3607528 10.1016/0361-9230(87)90129-8

[6] Murphy, C., Gilmore, M. M., Seery, C. S., Salmon, D. P. & Lasker, B. R. Olfactory thresholds are associated with degree of dementia in Alzheimer’s disease. *Neurobiol. Aging* **11**, 465–469 (1990). 2381506 10.1016/0197-4580(90)90014-q

[7] Moberg, P. J. et al. Olfactory identification in elderly schizophrenia and Alzheimer’s disease. *Neurobiol. Aging* **18**, 163–167 (1997). 9258893 10.1016/s0197-4580(97)00015-8

[8] Braak, H. et al. Staging of brain pathology related to sporadic Parkinson’s disease. *Neuobiol. Aging* **24**, 197–211 (2003). 10.1016/s0197-4580(02)00065-9 12498954

[9] Murray, H. C. et al. The unfolded protein response is activated in the olfactory system in Alzheimer’s disease. *Acta Neuropathol. Commun.* **8**, 109 (2020). 32665027 10.1186/s40478-020-00986-7 PMC7362534

[10] Stevenson, T. J. et al. synuclein inclusions are abundant in non-neuronal cells in the anterior olfactory nucleus of the Parkinson’s disease olfactory bulb. *Sci. Rep.* **10**, 6682 (2020). 32317654 10.1038/s41598-020-63412-x PMC7174302

[11] Ekström, I. et al. Smell loss predicts mortality risk regardless of dementia conversion. *J. Am. Geriatr. Soc.* **65**, 1238–1243 (2017). 28326534 10.1111/jgs.14770

[12] Roh, D. et al. The association between olfactory dysfunction and cardiovascular disease and its risk factors in middle-aged and older adults. *Sci. Rep.* **11**, 1248 (2021). 33441955 10.1038/s41598-020-80943-5 PMC7806612

[13] Papazian, E. J. & Pinto, J. M. Olfactory loss and aging: connections with health and well-being. *Chem. Senses* **46**, bjab045 (2021). 34673938 10.1093/chemse/bjab045

[14] Lang, J. *Clinical Anatomy of the Nose, Nasal Cavity and Paranasal Sinuses* (Thieme Medical Publishers, 1989).

[15] Leblanc, A. Olfactory nerves (I). In: *The cranial Nerves* (Springer Berlin Heidelberg, 1995).

[16] Leblanc, A. Olfactory nerves (I). In: *Encephalo-Peripheral Nervous System* (Springer Berlin Heidelberg, 2004).

[17] Moran, D. T., Rowley, J. C., Jafek, B. W. & Lovell, M. A. The fine structure of the olfactory mucosa in man. *J. Neurocytol.* **11**, 721–746 (1982). 7143026 10.1007/BF01153516

[18] Morrison, E. E. & Costanzo, R. M. Morphology of the human olfactory epithelium. *J. Comp. Neurol.* **297**, 1–13 (1990). 2376627 10.1002/cne.902970102

[19] Morrison, E. E. & Costanzo, R. M. Morphology of olfactory epithelium in humans and other vertebrates. *Microsc. Res. Technol.* **23**, 49–61 (1992). 10.1002/jemt.1070230105 1392071

[20] Salazar, I., Sanchez-Quinteiro, P. A., Barrios, W., López Amado, M. & Vega, J. A. Anatomy of the olfactory mucosa. *Handb. Clin. Neurol.* **164**, 47–65 (2019). 31604563 10.1016/B978-0-444-63855-7.00004-6

[21] von Brunn, A. Beiträge zur mikroskopischen Anatomie der menschlichen Nasenhöhle. *Archiv f. mikrosk. Anatomie* **39**, 632–651 (1892).

[22] Garcia, G. J. et al. Dosimetry of nasal uptake of water-soluble and reactive gases: a first study of interhuman variability. *Inhal. Toxicol.* **21**, 607–618 (2009). 19459775 10.1080/08958370802320186

[23] Buck, L. & Axel, R. A novel multigene family may encode odorant receptors: a molecular basis for odor recognition. *Cell* **65**, 175–187 (1991). 1840504 10.1016/0092-8674(91)90418-x

[24] Khan, M. et al. Anatomical barriers against SARS-CoV-2 neuroinvasion at vulnerable interfaces visualized in deceased COVID-19 patients. *Neuron* **110**, 3919–3935 (2022). 36446381 10.1016/j.neuron.2022.11.007 PMC9647025

[25] Vasvári, G., Reisch, R. & Patonay, L. Surgical anatomy of the cribriform plate and adjacent areas. *Minim. Invasive Neurosurg.* **48**, 25–33 (2005). 15747213 10.1055/s-2004-830180

[26] Cardali, S. et al. Microsurgical anatomic features of the olfactory nerve: relevance to olfaction preservation in the pterional approach. *Neurosurgery* **57**, 17–21 (2005). 15987566 10.1227/01.neu.0000144844.72403.7b

[27] Soriano, R. M., Solares, C. A., Pradilla, G. & DelGaudio, J. M. Endoscopic study of the distribution of olfactory filaments: a cadaveric study. *Am. J. Rhinol. Allergy* **35**, 226–233 (2021). 32762251 10.1177/1945892420948784

[28] López-Elizalde, R. et al. Anatomy of the olfactory nerve: a comprehensive review with cadaveric dissection. *Clin. Anat.* **31**, 109–117 (2018). 29088516 10.1002/ca.23003

[29] Crespo, C., Liberia, T., Blasco-Ibáñez, J. M., Nácher, J. & Varea, E. Cranial pair I: the olfactory nerve. *Anat. Rec.* **302**, 405–427 (2019). 10.1002/ar.23816 29659152

[30] Holbrook, E. H., Wu, E., Curry, W. T., Lin, D. T. & Schwob, J. E. Immunohistochemical characterization of human olfactory tissue. *Laryngoscope* **121**, 1687–1701 (2011). 21792956 10.1002/lary.21856 PMC3181071

[31] Nakashima, T., Kimmelman, C. P. & Snow, J. B. Olfactory marker protein in the human olfactory pathway. *Arch. Otolaryngol.* **111**, 294–297 (1985). 3885925 10.1001/archotol.1985.00800070046004

[32] Smith, R. L., Baker, H., Kolstad, K., Spencer, D. D. & Greer, C. A. Localization of tyrosine hydroxylase and olfactory marker protein immunoreactivities in the human and macaque olfactory bulb. *Brain Res.* **548**, 140–148 (1991). 1678294 10.1016/0006-8993(91)91115-h

[33] Maresh, A., Rodriguez Gil, D., Whitman, M. C. & Greer, C. A. Principles of glomerular organization in the human olfactory bulb—implications for odor processing. *PLoS ONE* **3**, e2640 (2008). 18612420 10.1371/journal.pone.0002640 PMC2440537

[34] Zapiec, B. et al. A ventral glomerular deficit in Parkinson’s disease revealed by whole olfactory bulb reconstruction. *Brain* **140**, 2722–2736 (2017). 28969383 10.1093/brain/awx208 PMC5841151

[35] Smith, R. L., Baker, H. & Greer, C. A. Immunohistochemical analyses of the human olfactory bulb. *J. Comp. Neurol.* **333**, 519–530 (1993). 7690371 10.1002/cne.903330405

[36] Mombaerts, P. et al. Visualizing an olfactory sensory map. *Cell* **87**, 675–686 (1996). 8929536 10.1016/s0092-8674(00)81387-2

[37] Treloar, H. B., Feinstein, P., Mombaerts, P. & Greer, C. A. Specificity of glomerular targeting by olfactory sensory axons. *J. Neurosci.* **22**, 2469–2477 (2002). 11923411 10.1523/JNEUROSCI.22-07-02469.2002 PMC6758332

[38] Low, V. F. & Mombaerts, P. Odorant receptor proteins in the mouse main olfactory epithelium and olfactory bulb. *Neuroscience* **344**, 167–177 (2017). 28057532 10.1016/j.neuroscience.2016.12.044

[39] Zapiec, B. & Mombaerts, P. The zonal organization of odorant receptor gene choice in the main olfactory epithelium of the mouse. *Cell Rep.* **30**, 4220–4234 (2020). 32209480 10.1016/j.celrep.2020.02.110

[40] Malnic, B., Hirono, J., Sato, T. & Buck, L. B. Combinatorial receptor codes for odors. *Cell* **96**, 713–723 (1999). 10089886 10.1016/s0092-8674(00)80581-4

[41] Saraiva, L. R. et al. Hierarchical deconstruction of mouse olfactory sensory neurons: from whole mucosa to single-cell RNA-seq. *Sci. Rep.* **5**, 18178 (2015). 26670777 10.1038/srep18178 PMC4680959

[42] Richard, M. B., Taylor, S. R. & Greer, C. A. Age-induced disruption of selective olfactory bulb synaptic circuits. *Proc. Natl. Acad. Sci. USA* **107**, 15613–15618 (2010). 20679234 10.1073/pnas.1007931107 PMC2932573

[43] Barnes, I. H. et al. Expert curation of the human and mouse olfactory receptor gene repertoires identifies conserved coding regions split across two exons. *BMC Genom.* **21**, 196 (2020). 10.1186/s12864-020-6583-3 PMC7055050 32126975

[44] Ronneberger, O., Fischer, P. & Brox, T. U-Net: convolutional networks for biomedical image segmentation. In: Navab, N., Hornegger, J., Wells, W., Frangi, A. (eds) *Medical Image Computing and Computer-Assisted Intervention*. *MICCAI 2015. Lecture Notes in Computer Science*, 9351 (Springer, 2015). 10.1007/978-3-319-24574-4_28.

[45] Nakashima, T., Kimmelman, C. P. & Snow, J. B. Structure of human fetal and adult olfactory neuroepithelium. *Arch. Otolaryngol.* **110**, 641–646 (1984). 6477257 10.1001/archotol.1984.00800360013003

[46] Paik, S. I., Lehman, M. N., Seiden, A. M., Duncan, H. J. & Smith, D. V. Human olfactory biopsy: the influence of age and receptor distribution. *Arch. Otolaryngol. Head Neck Surg.* **118**, 731–738 (1992). 1627295 10.1001/archotol.1992.01880070061012

[47] Nibu, K. et al. Olfactory neuron-specific expression of NeuroD in mouse and human nasal mucosa. *Cell Tissue Res.* **298**, 405–414, https://rdcu.be/daxzF (1999). 10639731 10.1007/s004419900098

[48] Shimizu, S. et al. A histochemical analysis of neurofibrillary tangles in olfactory epithelium, a study based on an autopsy case of juvenile Alzheimer’s disease. *Acta Histoch. Cytoch.* **55**, 93–98 (2022). 10.1267/ahc.22-00048 PMC9253500 35821750

[49] Barber, P. C. Ulex europeus agglutinin I binds exclusively to primary olfactory neurons in the rat nervous system. *Neuroscience* **30**, 1–9 (1989). 2747905 10.1016/0306-4522(89)90348-5

[50] Nagao, M., Oka, N., Kamo, H., Akiguchi, I. & Kimura, J. Ulex europaeus I and glycine max bind to the human olfactory bulb. *Neurosci. Lett.* **164**, 221–224 (1993). 8152605 10.1016/0304-3940(93)90896-s

[51] Margolis, F. L. A brain protein unique to the olfactory bulb. *Proc. Natl Acad. Sci. USA* **69**, 1221–1224 (1972). 4624756 10.1073/pnas.69.5.1221 PMC426668

[52] Nakashima, T., Tanaka, M., Inamitsu, M. & Uemura, T. Immunohistopathology of variations of human olfactory mucosa. *Eur. Arch. Otorhinolaryngol.* **248**, 370–375 (1991). 1930988 10.1007/BF00169031

[53] Marstal, K., Berendsen. F., Staring, M. & Klein, S. SimpleElastix: a user-friendly, multi-lingual library for medical image registration. In: *Proc. IEEE Conference on Computer Vision and Pattern Recognition Workshops*, 574–582 ((Las Vegas, 2016).

[54] Malandain, G., Bardinet, E., Nelissen, K. & Vanduffel, W. Fusion of autoradiographs with an MR volume using 2-D and 3-D linear transformations. *NeuroImage* **23**, 111–127 (2004). 15325358 10.1016/j.neuroimage.2004.04.038

[55] Ahrens, J. P., Geveci, B. & Law, C. C. ParaView: an end-user tool for large-data visualization. In: Hansen, C. D., Johnson, C. R. (eds.) *The Visualization Handbook* 717–731 (Elsevier Butterworth-Heinemann, 2005).

[56] Ayachit, U. *The ParaView Guide: a Parallel Visualization Application* (Kitware, Inc., 2015).

[57] Maitin-Shepard, J. et al. google/neuroglancer: (v2.23). *Zenodo* https://zenodo.org/records/5573294 (2021).

[58] Favre, J. J., Chaffanjon, P., Passagia, J. G. & Chirossel, J. P. Blood supply of the olfactory nerve. Meningeal relationships and surgical relevance. *Surg. Radiol. Anat.* **17**, 133–138 (1995). 7482150 10.1007/BF01627573

[59] Hendrix, P. et al. Arterial supply of the upper cranial nerves: a comprehensive review. *Clin. Anat.* **27**, 1159–1166 (2014). 24863843 10.1002/ca.22415

[60] Schindelin, J. et al. Fiji: an open-source platform for biological-image analysis. *Nat. Methods* **9**, 676–682 (2012). 22743772 10.1038/nmeth.2019 PMC3855844

[61] Solle, P. Erosion and dilation. In: *Morphological Image Analysis*. 63–103 (Springer Berlin Heidelberg, 2004).

[62] Barrios, A. W., Núñez, G., Sánchez Quinteiro, P. & Salazar, I. Anatomy, histochemistry, and immunohistochemistry of the olfactory subsystems in mice. *Front. Neuroanat.* **8**, 63 (2014). 25071468 10.3389/fnana.2014.00063 PMC4094888

[63] Engström, H. & Bloom, G. The structure of the olfactory region in man. *Acta Otolaryngol.* **43**, 11–21 (1953). 13039999 10.3109/00016485309119814

[64] Naessen, R. The identification and topographical localisation of the olfactory epithelium in man and other mammals. *Acta Otolaryngol.* **70**, 51–57 (1970). 4917513 10.3109/00016487009181858

[65] Naessen, R. An enquiry on the morphological characteristics and possible changes with age in the olfactory region of man. *Acta Otolaryngol.* **71**, 49–62 (1971). 5100075 10.3109/00016487109125332

[66] Holbrook, E. H., Leopold, D. A. & Schwob, J. E. Abnormalities of axon growth in human olfactory mucosa. *Laryngoscope* **115**, 2144–2154 (2005). 16369158 10.1097/01.MLG.0000181493.83661.CE

[67] Escada, P. Localization and distribution of human olfactory mucosa in the nasal cavities. *Acta Med. Port.* **26**, 200–207 (2013). 23815832

[68] Holbrook, E. H., Rebeiz, L. & Schwob, J. E. Office-based olfactory mucosa biopsies. *Int. Forum. Allergy Rhinol.* **6**, 646–653 (2016). 26833660 10.1002/alr.21711 PMC4921276

[69] Fitzek, M. et al. Integrated age-related immunohistological changes occur in human olfactory epithelium and olfactory bulb. *J. Comp. Neurol.* **530**, 2154–2175 (2022). 35397118 10.1002/cne.25325 PMC9232960

[70] Oliva, A. D. et al. Aging-related olfactory loss is associated with olfactory stem cell transcriptional alterations in humans. *J. Clin. Investig.* **132**, e155506 (2022). 34990409 10.1172/JCI155506 PMC8843745

[71] Khan, M. et al. Visualizing in deceased COVID-19 patients how SARS-CoV-2 attacks the respiratory and olfactory mucosae but spares the olfactory bulb. *Cell* **184**, 5932–5949 (2021). 34798069 10.1016/j.cell.2021.10.027 PMC8564600

[72] Clijsters, M. et al. Protocol for postmortem bedside endoscopic procedure to sample human respiratory and olfactory cleft mucosa, olfactory bulbs, and frontal lobe. *STAR Protoc.* **5**, 102831 (2024). 38277268 10.1016/j.xpro.2023.102831 PMC10837096

[73] Tanos, T. et al. Isolation of putative stem cells present in human adult olfactory mucosa. *PLoS One* **12**, e0181151 (2017). 28719644 10.1371/journal.pone.0181151 PMC5515430

[74] Nishijima, H. et al. Rapid fluorescent vital imaging of olfactory epithelium. *iScience* **25**, 104222 (2022). 35494237 10.1016/j.isci.2022.104222 PMC9046240

[75] Pinna, F. D., Ctenas, B., Weber, R., Saldiva, P. H. & Voegels, R. L. Olfactory neuroepithelium in the superior and middle turbinates: which is the optimal biopsy site. *Int. Arch. Otorhinolaryngol.* **17**, 131–138 (2013). 25992005 10.7162/S1809-97772013000200004 PMC4423289

[76] Kachramanoglou, C., Law, S., Andrews, P., Li, D. & Choi, D. Culture of olfactory ensheathing cells for central nerve repair: the limitations and potential of endoscopic olfactory mucosal biopsy. *Neurosurgery* **72**, 170–178 (2013). 23149968 10.1227/NEU.0b013e31827b99be

[77] Garcia, E. C. et al. Safety and efficacy of superior turbinate biopsies as a source of olfactory epithelium appropriate for morphological analysis. *Eur. Arch. Otorhinolaryngol.* **277**, 483–492 (2020). 31734722 10.1007/s00405-019-05728-7

[78] Garcia, E. C. et al. Biopsy of the olfactory epithelium from the superior nasal septum: is it possible to obtain neurons without damaging olfaction? *Braz. J. Otorhinolaryngol.* **88**, 787–793 (2022). 34144902 10.1016/j.bjorl.2021.05.008 PMC9483995

[79] Bressel, O. C., Khan, M. & Mombaerts, P. Linear correlation between the number of olfactory sensory neurons expressing a given mouse odorant receptor gene and the total volume of the corresponding glomeruli in the olfactory bulb. *J. Comp. Neurol.* **524**, 199–209 (2016). 26100963 10.1002/cne.23835 PMC4758392

[80] Low, V. et al. Visualizing the human olfactory projection and ancillary structures in a 3D reconstruction. *Zenodo* https://zenodo.org/records/7865523 (2023). 10.1038/s42003-024-07017-4 PMC11549439 39516237

