# Supplementary material for: Visualizing the human olfactory projection and ancillary structures in a 3D reconstruction
Source: Commun Biol. 2024 Nov 8;7:1467. doi: 10.1038/s42003-024-07017-4 (PMC11549439; doi:10.1038/s42003-024-07017-4)
Supplement: Supplementary file 1 — Supplementary Information [file 42003_2024_7017_MOESM1_ESM.pdf]

Supplementary Figures

Visualizing the human olfactory projection and ancillary structures in a 3D reconstruction

Victoria F. Low[1]
Chinchien Lin[2]
Shan Su[2]
Mahyar Osanlouy[2]
Mona Khan[3]
Soroush Safaei[2]
Gonzalo Maso Talou[2]
Maurice A. Curtis[1,4]
Peter Mombaerts[3,4]

[1]Department of Anatomy and Medical Imaging, The University of Auckland, Auckland, New Zealand
[2]Auckland Bioengineering Institute, The University of Auckland, Auckland, New Zealand
[3]Max Planck Research Unit for Neurogenetics, Frankfurt, Germany
[4]These authors contributed equally

Supplementary Figure 1 | Brightfield images of coronal sections of the six olfactory *en-bloc* specimens.

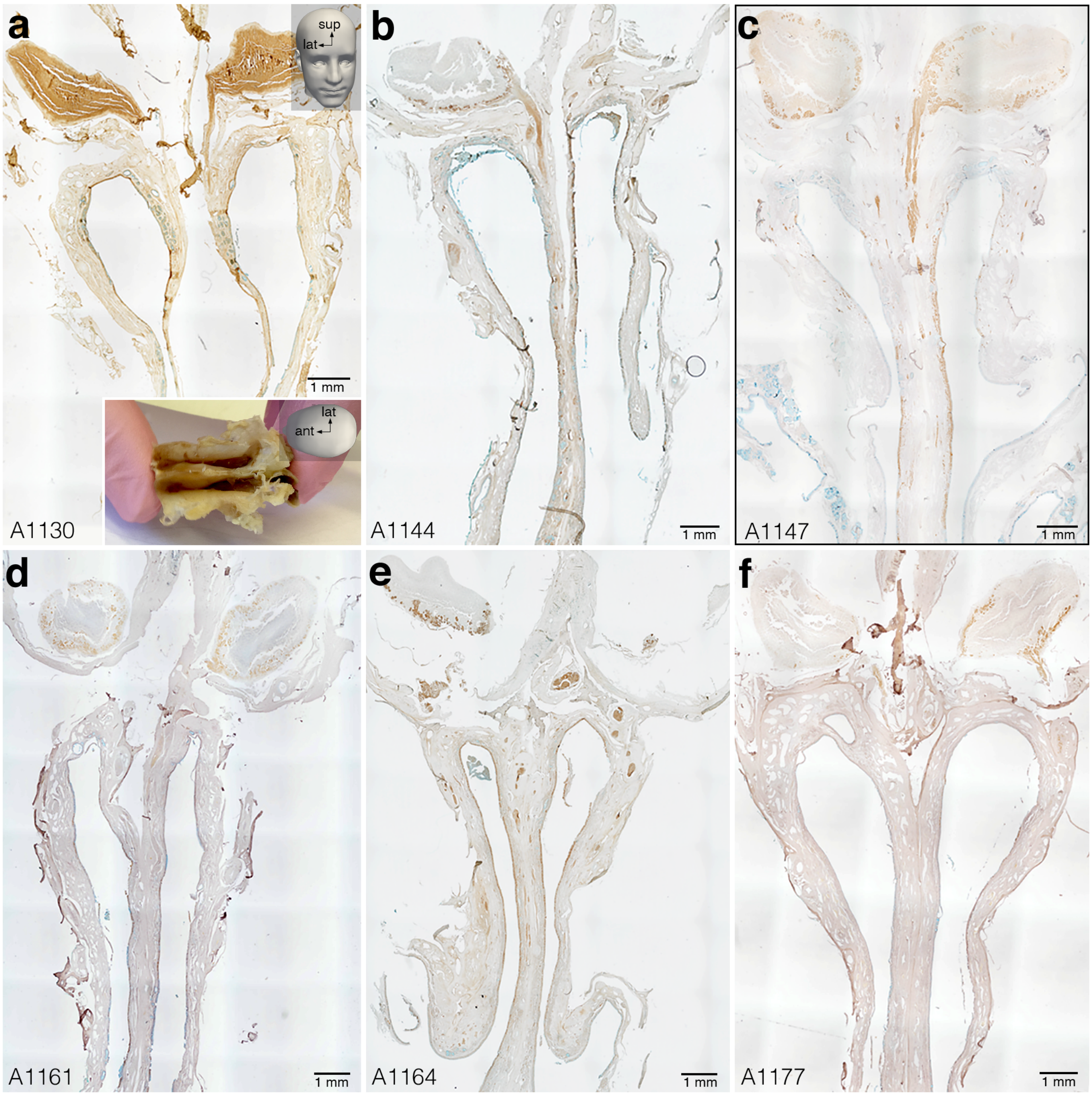

The 10 µm coronal sections were stained chromogenically with an antibody against OMP and with Alcian blue (blue, for mucins) and Hematoxylin (red, for nuclei). Tiled scans were taken under a brightfield microscope. The panels are organized according to the numbers of the cadavers, corresponding to the chronological order in which the bodies were received at The University of Auckland through the Human Body Bequest Programme. **a** Case A1130 was overprocessed. The lamina propria had separated from the nasal septum. There was immunoreactivity for OMP, but background signal was excessive. The inset shows a photo of the specimen after extraction and held between the thumb and index finger of V.F.L; the view is on the crista galli (superior face), the inferior face is not visible, and the olfactory bulbs are hidden by the shadows of the deep grooves. ant, anterior; lat, lateral; sup, superior. **b** Case A1144 had a damaged left olfactory bulb (visible on the right in this image). The nasal epithelium was thin and the lamina propria appeared overprocessed. **c** Case A1147 had the best anatomical preservation and immunoreactivity for OMP. The olfactory bulbs remained intact, the nasal epithelium sustained minimal damage during extraction, and the processing was optimal. This case was selected for 3D reconstruction. **d** The nasal septum of case A1161 was partially detached from the lamina propria, most likely due to the physical manipulations. **e** Case A1164 had a damaged left olfactory bulb (visible on the right in this image) and a partially detached right olfactory bulb (visible on the left). The nasal epithelium and lamina propria were in good condition. **f** Case A1177 was well preserved, had intact olfactory bulbs, and was appropriately processed. But it had low OMP immunoreactivity on the right side (visible on the left in this image).

Supplementary Figure 2 | Section distribution in the specimen.

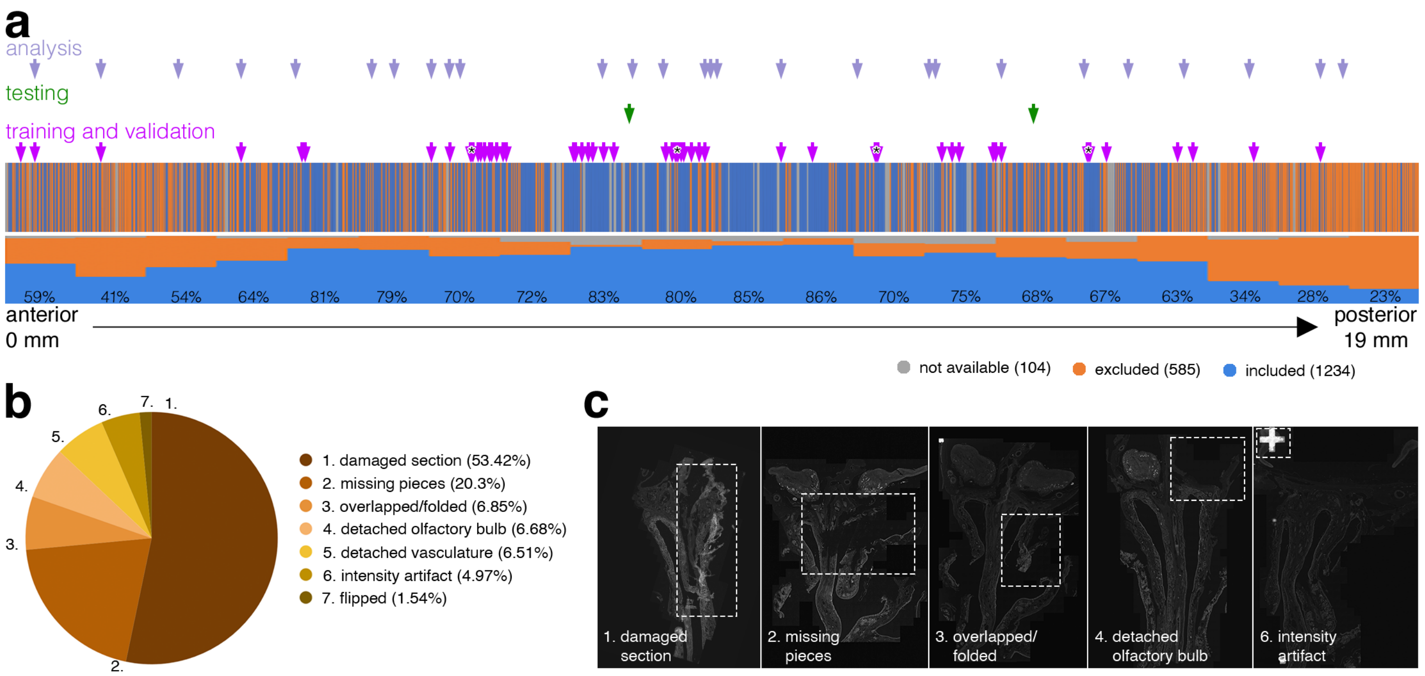

a Distribution of the 1923 10 µm coronal sections of case A1147. (Top panel) Gray vertical lines represent the 104 sections that were not available: 72 sections had been used already and 32 sections did not image properly during scanning. Orange vertical lines represent the 585 sections that were subsequently excluded. Blue vertical lines represent the 1234 sections that were retained and that make up the complete dataset. The arrows above the panel indicate the location of sections sampled for analysis such as counting olfactory sensory neurons and measuring epithelium thickness and length (lilac); of sections used for testing (green); and of sections used for training and validation (magenta). The four asterisks within the lilac arrows indicate the location of the initial 24 sections (four sets of six consecutive sections) in which all six structures of interest were segmented as ground truth. (Bottom panel) A bin represents 5% of the 1923 sections. The percentages in the blue bars represent the percentage of sections in a bin that were included. The gray and orange bars represent the fractions of sections in a bin that were, respectively, not available or subsequently excluded. **b** Pie chart of the criteria for exclusion of the 585 sections. Tissue damage was the most frequent cause for exclusion. **c** Examples of excluded sections. Numbers match the categories in b. The white dashed box indicates the relevant area that led the section to be excluded.

Supplementary Figure 3 | Summary of the training, validation, and testing of the convolutional neural networks.

	vasculature	olfactory sensory neurons	bone	glomeruli	olfactory bulb	nasal epithelium
**ground truth sections**						
**total (training/validation/testing)**	57 (45/10/2)	46 (38/6/2)	37 (29/6/2)	32 (25/5/2)	26 (20/4/2)	26 (20/4/2)
**training dataset**						
**GPU training (hours)**	20	22	45	49	6	28
**validation dataset**						
**Dice similarity coefficient**	0.712 ± 0.008	0.768 ± 0.013	0.862 ± 0.012	0.825 ± 0.012	0.978 ± 0.003	0.934 ± 0.007
**binary cross-entropy**	0.0209 ± 0.0009	0.0140 ± 0.0012	0.0139 ± 0.0007	0.0018 ± 0.0002	0.0017 ± 0.0001	0.0060 ± 0.0010
**testing dataset (#850; #1401)**						
**Dice similarity coefficient**	0.708; 0.808	0.682; 0.760	0.591; 0.885	0.482; 0.779	0.970; 0.967	0.879; 0.927
**binary cross-entropy**	0.0139; 0.0148	0.0147; 0.0173	0.0341; 0.0121	0.0012; 0.0017	0.0020; 0.0044	0.0139; 0.0053

Values for the Dice similarity coefficient and binary cross-entropy are given as mean ± SEM.

Supplementary Figure 4 | Comparison of the ground truth and convolutional neural network segmentations for olfactory sensory neurons.

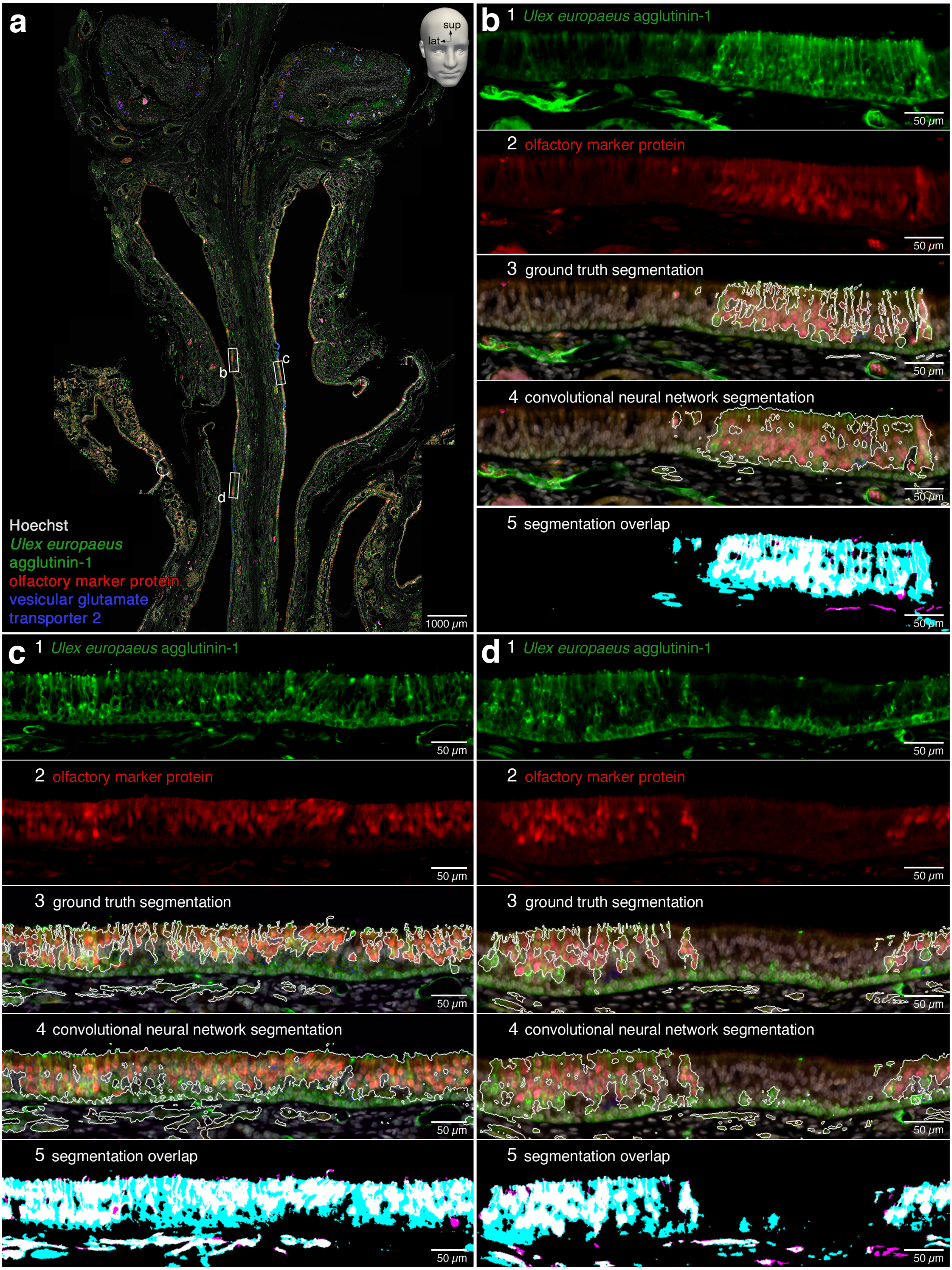

Supplementary Figure 4 | a Representative 10 µm coronal section (#912) stained fluorescently for Hoechst (gray), *Ulex europaeus* agglutinin-1 (green), olfactory marker protein (magenta), and vesicular glutamate transporter 2 (blue). Rectangles B-D are displayed at higher magnification. **b–d** 1, UEA1 (green) detects mature and immature OSNs, as well as basal cells. 2, OMP (red) detects maturing OSNs. 3, merge of Hoechst, UEA1, OMP, and VGLUT2 with the ground truth segmentation of OSNs displayed with a white outline. 4, merge of Hoechst, UEA1, OMP, and VGLUT2 with the CNN segmentation of OSNs displayed with a white outline. Note that basal cells are not included. 5, the overlap (white) of the ground truth segmentation (magenta) and the CNN segmentation (cyan) represents the agreement between these segmentations for OSNs. Where cyan is visible, the CNN segmentation has overpredicted, and where magenta is visible, the CNN has underpredicted. Finer details, including OSN dendrites and axons, are not properly detected, resulting in an overprediction in the CNN segmentation (more cyan) in comparison to the ground truth (white and magenta).

Supplementary Figure 5 | Surface areas, volumes, and interleaved sampling.

				complete dataset	interleaved sampling percentage error		
**olfactory sensory neurons**					1 in 4	1 in 16	1 in 32
	surface area in nasal epithelium (olfactory epithelium)						
		left		179.83 mm^2	1.04%	1.31%	0.02%
			lateral	82.86 mm^2	1.18%	1.02%	3.25%
			septal	96.97 mm^2	0.92%	3.25%	2.79%
		right		161.82 mm^2	0.68%	2.13%	0.08%
			lateral	81.64 mm^2	0.35%	5.50%	3.95%
			septal	80.18 mm^2	1.72%	1.24%	3.54%
		**total**		**341.65 mm^2**	**0.23%**	**1.70%**	**0.05%**
	volume						
		left		31.61 mm^3	0.78%	1.13%	0.97%
		right		29.82 mm^3	0.92%	1.19%	2.95%
		**total**		**61.43 mm^3**	**0.85%**	**0.00%**	**0.91%**
	volume in olfactory bulb						
		left		9.20 mm^3	0.71%	4.37%	4.29%
		right		9.70 mm^3	0.89%	1.54%	3.30%
		**total**		**18.90 mm^3**	**0.12%**	**1.30%**	**3.79%**
**olfactory bulb**							
	volume						
		left		66.32 mm^3	0.14%	0.88%	0.07%
		right		68.21 mm^3	0.72%	0.82%	1.30%
		**total**		**134.53 mm^3**	**0.28%**	**0.85%**	**0.67%**
**glomeruli**							
	volume						
		left		1.55 mm^3	0.22%	1.72%	6.61%
		right		1.34 mm^3	1.52%	0.62%	14.14%
		**total**		**2.89 mm^3**	**0.59%**	**1.21%**	**10.12%**

The surface areas of OSNs in the nasal epithelium in mm^2 are listed for the left and right nasal cavities and their total, and for the lateral and septal aspects of each cavity. The volumes in mm^3 are listed for the OSNs; the part of the OSNs within the olfactory bulbs; the olfactory bulbs; and the glomeruli. The absolute changes of the percentage error resulting from interleaved quantifications at sampling rates of 1 in 4, 1 in 16, and 1 in 32 are listed.

Supplementary Figure 6 | Interleaved sampling.

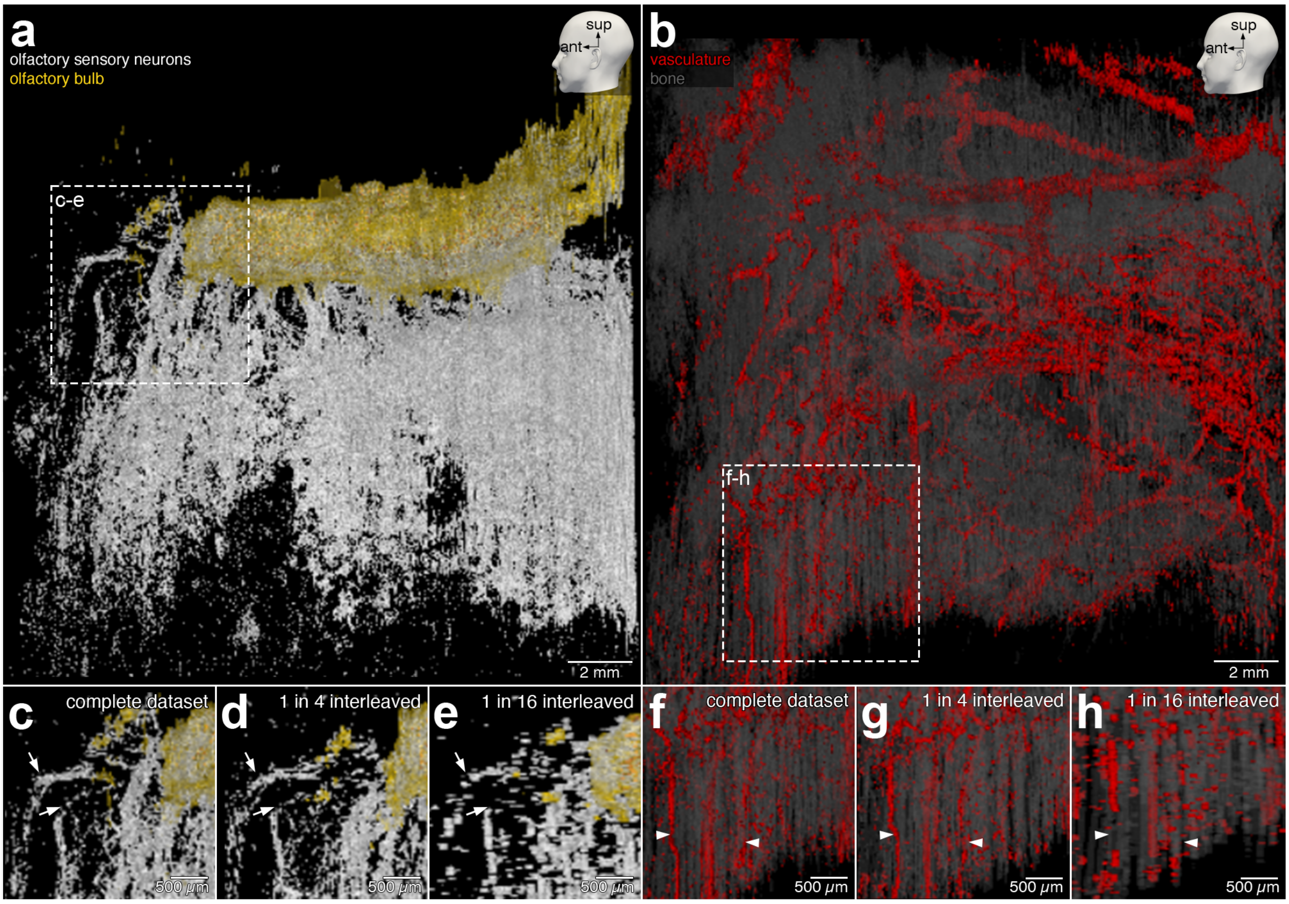

Images were taken in Neuroglancer. **a,b** Still views of the lateral aspect of the left nasal cavity, of the OSN and olfactory bulb segmentations (a) and the bone and vasculature segmentations (b). **c** The complete dataset was used for registration. White arrows point to two axon fascicles. **d** Registration at a sampling rate of 1 in 4 results in very little structural deformation of the axon fascicles. **e** Registration at a sampling rate of 1 in 16 rate results in a loss of continuity of finer details. **f** The complete dataset was used for registration. White arrowheads point to two blood vessels. **g** Registration at a sampling rate of 1 in 4 is still compatible with a smooth registration. **h** Registration at a sampling rate of a 1 in 16 is insufficient for a smooth registration. The two blood vessels indicated with white arrowheads are incomplete.
