# Supplementary material for: Visualizing the human olfactory projection and ancillary structures in a 3D reconstruction
Source: Commun Biol. 2024 Nov 8;7:1467. doi: 10.1038/s42003-024-07017-4 (PMC11549439; doi:10.1038/s42003-024-07017-4)
Supplement: Supplementary file 2 — Description of Additional Supplementary Files [file 42003_2024_7017_MOESM2_ESM.pdf]

# Description of Additional Supplementary Files

**File name:** Supplementary Movie 1

**Description:** Graphical animation of the 3D reconstruction- The animation begins with a fly-through of the registered fluorescently stained coronal sections, transitions into overlays of the CNN segmentations, and is followed by stacking the CNN segmentations to build a 3D visualization. This 3D reconstruction is then sliced through the left nasal cavity and opened, akin to opening a book, to expose a sideways view of the lateral and septal aspects of the nasal epithelium and the arrangement of glomeruli within the left olfactory bulb. The proximity of olfactory axon fascicles and arteries can be viewed. Fila olfactoria can be followed traversing ipsilaterally the cribriform plate toward the olfactory bulb. Adjusting the opacity of different segmentations as well as rotating the 3D reconstruction allow for a view of olfactory sensory neuron axons innervating the olfactory bulb and the dense vascularization of the olfactory projection. A 1 mm-thick virtual coronal slice is rotated to illustrate the interactions and connections between the various structures. Still views were captured from this movie at timepoints that are indicated next to the clapperboards in the panels of Figures 6 and 7.
