# Supplementary material for: Visualizing the human olfactory projection and ancillary structures in a 3D reconstruction
Source: Commun Biol. 2024 Nov 8;7:1467. doi: 10.1038/s42003-024-07017-4 (PMC11549439; doi:10.1038/s42003-024-07017-4)
Supplement: Supplementary file 4 — Reporting Summary [file 42003_2024_7017_MOESM4_ESM.pdf]

nature portfolio

Corresponding author(s): Peter Mombaerts

Last updated by author(s): 29 September 2024

Reporting Summary

Nature Portfolio wishes to improve the reproducibility of the work that we publish. This form provides structure for consistency and transparency in reporting. For further information on Nature Portfolio policies, see our Editorial Policies and the Editorial Policy Checklist.

Statistics

For all statistical analyses, confirm that the following items are present in the figure legend, table legend, main text, or Methods section.

n/a	Confirmed	
☐	☒	The exact sample size (n) for each experimental group/condition, given as a discrete number and unit of measurement
☐	☒	A statement on whether measurements were taken from distinct samples or whether the same sample was measured repeatedly
☐	☒	The statistical test(s) used AND whether they are one- or two-sided<br>*Only common tests should be described solely by name; describe more complex techniques in the Methods section.*
☒	☐	A description of all covariates tested
☒	☐	A description of any assumptions or corrections, such as tests of normality and adjustment for multiple comparisons
☐	☒	A full description of the statistical parameters including central tendency (e.g. means) or other basic estimates (e.g. regression coefficient) AND variation (e.g. standard deviation) or associated estimates of uncertainty (e.g. confidence intervals)
☒	☐	For null hypothesis testing, the test statistic (e.g. F, t, r) with confidence intervals, effect sizes, degrees of freedom and P value noted<br>*Give P values as exact values whenever suitable.*
☒	☐	For Bayesian analysis, information on the choice of priors and Markov chain Monte Carlo settings
☒	☐	For hierarchical and complex designs, identification of the appropriate level for tests and full reporting of outcomes
☒	☐	Estimates of effect sizes (e.g. Cohen's d, Pearson's r), indicating how they were calculated

Our web collection on statistics for biologists contains articles on many of the points above.

Software and code

Policy information about availability of computer code

Data collection

Nikon Eclipse NiE microscope Nikon
NIS elements AR Version 5.21.03 Nikon
Pannoramic MIDI II fluorescence digital slide scanner 3DHISTECH https://www.3dhistech.com
CaseViewer Version 2.4.0.119028 3DHISTECH https://www.3dhistech.com/

Data analysis

Adobe Suite: Acrobat Pro 2022, Photoshop 2022, Premiere Pro 2022 Adobe Adobe Creative Cloud
Amira software Version 6.5.0 Thermo Fisher Scientific https://www.thermofisher.com
Excel 2022 Microsoft Microsoft
Fiji ImageJ2 version 2.3.0/1.53n Schindelin et al., 2012 https://imagej.net/
Neuroglancer Maitin-Shepard et al., 2021 https://github.com/google/neuroglancer
ParaView Version 5.10.1 Ahrens et al., 2005 Ayachit et al., 2015 https://www.paraview.org
Python 3 Python Software Foundation https://www.python.org/
SimpleElastix Marstal et al., 2016 https://simpleelastix.github.io/
Tensorflow v2 Google https://www.tensorflow.org/
U-Net architecture Ronneberger et al., 2015 https://lmb.informatik.uni-freiburg.de

Hardware/Infrastructure
Auckland Bioengineering Institute High performance computing https://www.auckland.ac.nz/en/abi.html
eResearch drive The University of Auckland https://www.eresearch.auckland.ac.nz/
Globus University of Chicago https://www.globus.org

Nectar research cloud Australian Research Data Commons https://ardc.edu.au/
New Zealand eScience Infrastructure The University of Auckland https://www.nesi.org.nz/
Precision 7920 tower workstation (Intel Xeon Gold 5218 CPU (2.30GHz, 2295 Mhz, 16 core, 32 logical processors), NIVIDIA Quadro P6000 24.0GB, and 128 GB RAM) DELL https://www.dell.com

Original code https://doi.org/10.5281/zenodo.7865523

For manuscripts utilizing custom algorithms or software that are central to the research but not yet described in published literature, software must be made available to editors and reviewers. We strongly encourage code deposition in a community repository (e.g. GitHub). See the Nature Portfolio guidelines for submitting code & software for further information.

Data

Policy information about availability of data

All manuscripts must include a data availability statement. This statement should provide the following information, where applicable:
- Accession codes, unique identifiers, or web links for publicly available datasets
- A description of any restrictions on data availability
- For clinical datasets or third party data, please ensure that the statement adheres to our policy

Original code: https://doi.org/10.5281/zenodo.7865523

Research involving human participants, their data, or biological material

Policy information about studies with human participants or human data. See also policy information about sex, gender (identity/presentation), and sexual orientation and race, ethnicity and racism.

Reporting on sex and gender	yes
Reporting on race, ethnicity, or other socially relevant groupings	n/a
Population characteristics	n/a
Recruitment	n/a
Ethics oversight	Postmortem en-bloc specimens were collected at the Human Anatomy Lab of The University of Auckland through the Human Body Bequest Programme, which is governed by the New Zealand Police, and in compliance with the Human Tissue Act 2008 from the New Zealand Parliament.

Note that full information on the approval of the study protocol must also be provided in the manuscript.

Field-specific reporting

Please select the one below that is the best fit for your research. If you are not sure, read the appropriate sections before making your selection.

☒ Life sciences ☐ Behavioural & social sciences ☐ Ecological, evolutionary & environmental sciences

For a reference copy of the document with all sections, see nature.com/documents/nr-reporting-summary-flat.pdf

Life sciences study design

All studies must disclose on these points even when the disclosure is negative.

Sample size	n=6 Primary olfactory pathway en-bloc specimens extracted from embalmed human cadavers. A1130, A1144, A1147, A1161, A1164, A1177. Human Body Bequest Programme Human Anatomy Lab, The University of Auckland
Data exclusions	n=5 excluded for quality reasons described in the ms
Replication	n=1
Randomization	n/a
Blinding	n/a

Reporting for specific materials, systems and methods

We require information from authors about some types of materials, experimental systems and methods used in many studies. Here, indicate whether each material, system or method listed is relevant to your study. If you are not sure if a list item applies to your research, read the appropriate section before selecting a response.

Materials & experimental systems

n/a	Involved in the study
☐	☒ Antibodies
☒	☐ Eukaryotic cell lines
☒	☐ Palaeontology and archaeology
☒	☐ Animals and other organisms
☒	☐ Clinical data
☒	☐ Dual use research of concern
☒	☐ Plants

Methods

n/a	Involved in the study
☒	☐ ChIP-seq
☒	☐ Flow cytometry
☒	☐ MRI-based neuroimaging

Antibodies

Antibodies used

Anti-Olfactory Marker Protein antibody, unconjugated, goat FUJIFILM Wako Shibayagi Cat#019-22291-WAKO; RRID: AB_664696
Anti-Vesicular Glutamate Transporter 2 antibody, unconjugated, guinea pig Frontier Science Cat#VGluT2-GP-Af670-1

Alexa Fluor 647-AffiniPure donkey anti-Guinea pig IgG (H+L) (min X Bov,Ck,Gt,Sy Hms,Hrs,Hu,Ms,Rb,Rat,Shp Sr Prot) antibody Jackson ImmunoResearch Laboratories Cat#706-605-148; RRID: AB_2340476
Alexa Fluor Plus 594 donkey anti-goat IgG (H+L) highly cross-adsorbed secondary antibody Thermo Fisher Scientific Cat#A32758; RRID: AB_2762828
Biotin-SP-AffiniPure donkey anti-Goat IgG (H+L) antibody Jackson ImmunoResearch Laboratories Cat#705-065-003; RRID: AB_2340396

Validation

Anti-Olfactory Marker Protein antibody: Peer-reviewed publications listed on manufacturer website
Anti-Vesicular Glutamate Transporter 2 antibody: Manufacturer website "Validated for use in Western Blotting, Immunohistochemistry for the detection of VGLUT2 and Peer-reviewed publications listed on manufacturer website
Ulex Europaeus Agglutinin-1 (UEA1) antibody: Peer-reviewed publications listed on manufacturer website

Plants

Seed stocks

n/a

Novel plant genotypes

n/a

Authentication

n/a
